# Variation in approaches to antimicrobial use surveillance in high-income secondary care settings: a systematic review

**DOI:** 10.1093/jac/dkab125

**Published:** 2021-04-24

**Authors:** Selina Patel, Arnoupe Jhass, Ann Slee, Susan Hopkins, Laura Shallcross

**Affiliations:** 1 Institute of Health Informatics, University College London, London, UK; 2 Research Department of Primary Care & Population Health, University College London, London, UK; 3 NHS England, London, UK; 4 Public Health England, London, UK

## Abstract

**Introduction:**

In secondary care, antimicrobial use (AMU) must be monitored to reduce the risk of antimicrobial resistance and infection-related complications. However, there is variation in how hospitals address this challenge, partly driven by each site’s level of digital maturity, expertise and resources available. This systematic review investigated approaches to measuring AMU to explore how these structural differences may present barriers to engagement with AMU surveillance.

**Methods:**

We searched four digital databases and the websites of relevant organizations for studies in high-income, inpatient hospital settings that estimated AMU in adults. Excluded studies focused exclusively on antiviral or antifungal therapies. Data were extracted data on 12 fields (study description, data sources, data extraction methods and professionals involved in surveillance). Proportions were estimated with 95% CIs.

**Results:**

We identified 145 reports of antimicrobial surveillance from Europe (63), North America (53), Oceania (14), Asia (13) and across more than continent (2) between 1977 and 2018. Of 145 studies, 47 carried out surveillance based on digital data sources. In regions with access to electronic patient records, 26/47 studies employed manual methods to extract the data. The majority of identified professionals involved in these studies were clinically trained (87/93).

**Conclusions:**

Even in regions with access to electronic datasets, hospitals rely on manual data extraction for this work. Data extraction is undertaken by healthcare professionals, who may have conflicting priorities. Reducing barriers to engagement in AMU surveillance requires investment in methods, resources and training so that hospitals can extract and analyse data already contained within electronic patient records.

## Introduction

Antimicrobial-resistant infections in humans are increasing.^1–3^ These infections are harder to treat and associated with worse health outcomes than infections that are susceptible to conventional therapies.^4^ One of the most well-documented associations with increases in resistance is antimicrobial use (AMU) in humans.^5^^,^^6^ In high-income settings, most AMU is regulated by healthcare professionals and although the majority of AMU is prescribed in primary care, there is a concentration of use in secondary care settings. The prevalence of hospital inpatients on antimicrobial therapy is around 30%^7^^,^^8^ and some 30% of these prescriptions may not be in line with guidelines for AMU.^9^^,^^10^ Antimicrobial stewardship (AMS) promotes and monitors judicious use of antimicrobials to preserve their effectiveness,^11^ but this requires access to information about the volume and type of AMU across the hospital, for example to benchmark patterns of prescribing between wards or specialties and target interventions to where they can have the greatest impact. When a patient is admitted to hospital for several days and their clinical care and antibiotic prescriptions are managed by several specialty care teams, large datasets are a pre-requisite to being able to benchmark effectively.

Previous work has focused on measuring the quantity (amount of antibiotics used) and quality (process indicators of use) of AMU, but hospitals employ a variety of different metrics, making it difficult to use them for national surveillance. Several studies have hypothesized the need for a standard set of AMU metrics to address this issue,^12–14^ but there has been little consideration of the feasibility of adopting these measures across different hospitals considering factors such as data availability and digital maturity. The data sources available for surveillance are usually determined by the local systems used for hospital administration and patient care, which may be electronic or paper-based. The data points recorded in these systems provide the level of detail available for AMU surveillance. The accessibility of these data is contingent on either resource to manually extract data from these systems, or in-house teams to support digital data extraction with data scientists or academic centre partnerships available to carry out analysis of these datasets. These practical considerations may, to some extent, be influencing the way hospitals engage with AMU surveillance. However, so far, systematic reviews of AMU surveillance in this setting have not explored differences in data sources and data collection to describe how this variation presents in AMU surveillance specifically.

The aim of this review is to summarize the different approaches that are used for surveillance of AMU among hospital inpatients in high-income countries and to explore how issues such as digital maturity, the availability of electronic prescribing or electronic health record (EHR) data and access to trained personnel might represent barriers to measuring AMU. This study is described following the guidance laid out in the Preferred Reporting Items for Systematic review and Meta-Analysis (PRISMA).^15^

## Methods

### Information sources

#### Database searches

The following databases were searched on 2 August 2018 for relevant articles: Medline (Ovid), Embase (Ovid), Cumulative Index to Nursing and Allied Health Literature (CINAHL) and the Cochrane Central Register of Controlled Trials. The search strategy, which was adapted from the work of Stanić Benić *et al.*^16^ comprised four concepts: antibiotics, utilization, measure and hospital. The search terms were developed in Medline (Table 1) and then adapted to the other three databases included in the search. There were no time restrictions placed on articles in this review.

**Table 1.** Medline (Ovid) search strategy

Search concept	Search terms
Antibiotic	1. Anti-Bacterial Agents/ad, dt, sd, tu, th, ut [Administration & Dosage, Drug Therapy, Supply & Distribution, Therapeutic Use, Therapy, Utilization]
	2. Antibiotic Prophylaxis/ec, mt, sn, td, ut [Economics, Methods, Statistics & Numerical Data, Trends, Utilization]
	3. (anti? biotic? or anti? microbial? or anti? bacterial?).ab, ti.
	4. 1 or 2 or 3
Utilization	5. Drug Prescriptions/
	6. Drug Utilization/
	7. “Drug Utilization Review”/cl, ec, mt, st, sn, td, ut [Classification, Economics, Methods, Standards, Statistics & Numerical Data, Trends, Utilization]
	8. ((anti? biotic? or anti? microbial?) adj3 (prescri* or consumption or utili? ation or usage or “use” or dispens* or sale?)).ab, ti.
	9. 5 or 6 or 7 or 8
Measurement	10. BENCHMARKING/cl, ec, mt, st, sn, td, ut [Classification, Economics, Methods, Standards, Statistics & Numerical Data, Trends, Utilization]
	11. (intervention adj5 (prescri* or stewardship or “use” or utili? ation or usage or consumption)).ab, ti
	12. ((anti? biotic? or anti? microbial?) adj4 (estimat* or quantif* or metric? or monitor* or surveillance or prevalence or survey or audit)).ab, ti.
	13. (electronic prescri* or e? prescri*).ab, ti.
	14. 10 or 11 or 12 or 13
Secondary care	15. Secondary Care/
	16. Hospitals/
	17. hospital*.ab, ti.
	18. 15 or 16 or 17
	19. 4 and 9 and 14 and 18

.ab, abstract; ti., title; adj3, indicates two words next to each other in any order with up to two words in between; /, indicates a Medical Subject Heading (MeSH); *, denotes any truncation; ?, denotes one character or no character. All other abbreviations are elaborated upon in [] brackets in the table.

#### Website searches

The websites of 33 relevant organizations were searched during the period 24–28 March 2018 (Table S1, available as Supplementary data at *JAC* Online). The initial list of potential organizations was identified from a list originally conceived by Stanić Benić *et al.*^16^ and then expanded based on discussion between three of the authors of this review. Surveillance reports identified from website searches were included at the full-text screening stage of the review to ensure we captured national surveillance programmes that are often only reported in the grey literature.

Due to the high number of publications identified in the searches, it was deemed unnecessary to review the bibliographies of included articles to screen further relevant studies.

### Study selection and data collection process

The articles identified by the searches were uploaded to Mendeley reference manager software and exported to web-based Eppi Reviewer 4 systematic review software for duplicate removal, title screening by a single author (S.P.) and then title and abstract screening by two independent reviewers in duplicate (S.P. and A.J.). Next, references were transferred to DistillerSR software. Thirty full-text articles were screened at a time by two reviewers (S.P. and A.J.) in duplicate until >90% agreement was reached on article inclusion/exclusion. The remaining full-text articles were screened and data extracted by a single reviewer (S.P.). Data fields were extracted for each included study using an electronic form, which was piloted prior to use.

### Data items

Data were extracted on the following (12 items):

- **Study** (study design, country, year of publication, setting, study primary objective and number of participants)
- **Approach used to measure AMU** (data source, method of collection, reason for data collection, who collected the data, level of detail in the data and data type).

### Eligibility criteria

Reports that separately reported data on AMU in high-income countries or territories (as categorized by the World Bank in 2018) for at least 100 inpatients >17 years old in secondary care were included in the review. When the country was not classified in the World Bank Country and Lending Groups, the article was included if the gross national income per capita was 12 056 USD or more.^17^ We expected to capture a range of cross-sectional, cohort, ecological, randomized controlled trial and quasi-experimental study designs that attempted to estimate AMU for a variety of purposes. We did not include protocols, conference abstracts, degree theses, trials of antimicrobial therapies or reviews of the literature. Surveillance studies that did not report the total number of participants (study denominator) or that focused exclusively on antiviral or antifungal therapies were excluded. Only studies that were available in the English language were included due to the language spoken by the authors of this study. Studies were not excluded on the basis of quality as the aim of the review was to describe the range of approaches that are being used to monitor AMU.

### Assessment of risk of bias in individual studies and across studies

This was a descriptive review of existing AMU surveillance approaches so a risk-of-bias assessment was not carried out. All approaches to AMU surveillance included in the final review must have estimated AMU for at least 100 inpatients in secondary care. This was to ensure that the approach could be applied as a surveillance tool.

### Outcomes

#### Primary outcomes

- Proportion of AMU surveillance based on electronic datasets

#### Secondary outcomes

- A description of the methods of data extraction implemented in surveillance
- A description of the different professionals involved in surveillance

### Data synthesis

The data underwent a descriptive synthesis to describe the methodologies employed to monitor AMU in secondary care and the frequency with which they have been used. The data were extracted from Distiller-SR as an Excel file and analysed using R statistical software.^18^ Study characteristics were described using proportions with estimated 95% CIs.

Prior to starting the review, this study was registered on the Prospective Register of Systematic Reviews (registration number: CRD42018103375).

## Results

**Figure 1.**
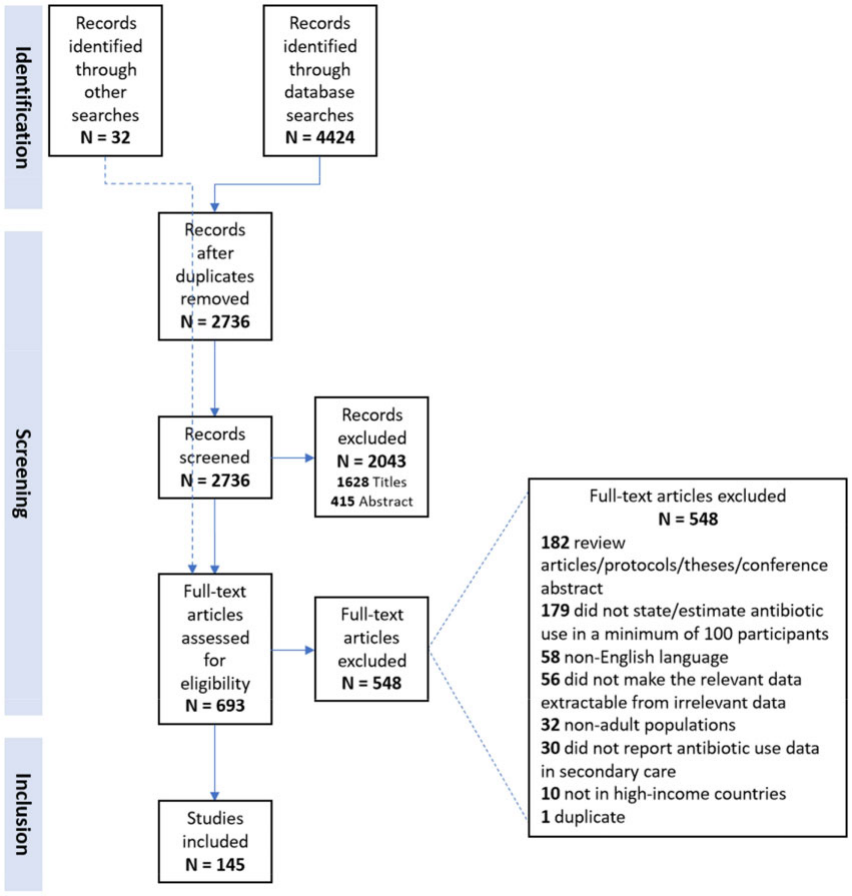
Screening of studies for inclusion in the review. This figure appears in colour in the online version of *JAC* and in black and white in the printed version of *JAC*.

The systematic review identified 2736 studies, 145 of which met study inclusion criteria (Figure 1). The studies included data from 40 high-income countries and the median year of publication was 2012 (range 1977–2018) (Table S2). Sixty-three studies (43%) were carried out in Europe, 53 (37%) in North America, 14 (10%) in Oceania, 13 (9%) in Asia and 2 across more than one continent (1%). In total, the 145 studies estimated AMU in 1 025 640 inpatients across 1966 hospital sites. Included hospitals ranged from 48 to 3000 beds. The hospital size was not stated for 54 studies. All hospital patients (excluding psychiatric and paediatric) were eligible for inclusion in surveillance in 47 studies, 80 studies monitored use among patients on wards/specialties or with an indication/diagnosis/contraindication of interest, 13 surveyed use of target antimicrobials only and 5 studies excluded patients on prophylactic regimens of antimicrobials.

### Data sources and data extraction for surveillance

There were 109 reports of surveillance that was carried out by manually extracting data from relevant sources (74%, 95% CI 66.6–80.8) (Table 2). Of these studies, 61 included information on the data source that was used. This showed that the majority of studies (31/61) manually extracted data from paper notes or by talking to clinicians. However, 16/61 manually extracted data from digital sources such as EHRs. Separately, 13 studies harnessed data through a combination of manually and digitally extracting the data. In 10/13 of these studies, only digital data sources were used (7%, 95% CI 2.7–10.8).

**Table 2.** Methods applied to extract data from different sources used in surveillance

Method of data extraction	Frequency of data extraction method employed (%, 95% CI)	Data source	Frequency of data source used (%, 95% CI)
Manual (3 counted twice)^a^	109 (74, 66.6–80.8)	Paper notes/prescriptions/talking to relevant clinicians	31 (21, 14.4–27.5)
		EHRs/prescriptions/pharmacy dispensing data/billing data/hospital census data	16 (11, 5.8–15.8)
		Combination of digital and paper/speaking to clinicians	14 (10, 4.8–14.2)
		Not specified or unclear	48 (32, 24.9–40.0)
Digital	19 (13, 7.5–18.2)	EHRs/electronic prescriptions/billing data/hospital census data/ routinely collected data for other reasons	18 (12, 6.9–17.4)
		Not specified	1 (<1, 0.0–2.0)
Manual and digital	13 (9, 4.2–13.3)	EHRs/prescriptions/pharmacy dispensing data/billing data/hospital census data	10 (7, 2.7–10.8)
		Combination of digital and paper/speaking to clinicians	2 (1, 0.0–3.2)
		Not specified or unclear	1 (<1, 0.0–2.0)
Not specified or unclear	7 (5, 1.3–8.2)	EHRs/electronic prescriptions/billing data/hospital census data/routinely collected data for other reasons	2 (1, 0.0–3.2)
		Not specified or unclear	5 (3, 0.5–6.3)

*N* = 148 (3 reports used different sources at different sites).

a Surveillance is counted more than once where more than one approach was applied across sites.

### Geographical distribution of digital surveillance

**Figure 2.**
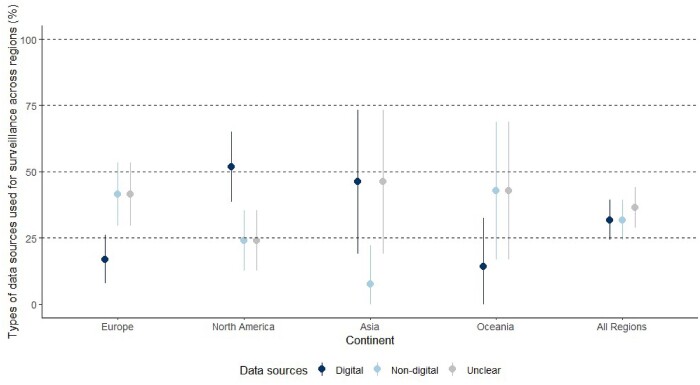
The types of data sources harnessed for surveillance, by region. Europe, *n* = 65; North America, *n* = 54; Asia, *n* = 13; Oceania, *n* = 14; all regions, *n* = 148 (3 reports used different sources at different sites). Intercontinental studies not shown: Europe/America, *n* = 1 unclear; Europe/Asia, *n* = 1 unclear. This figure appears in colour in the online version of *JAC* and in black and white in the printed version of *JAC*.

The overall proportion of surveillance approaches that harnessed digital data sources for surveillance was 47/148 (32%, 95% CI 24.3–39.3) and varied by region (Figure 2 and Table S3). In North America, 28/54 (52%, 95% CI 38.5–65.2) harnessed digital data sources for surveillance, whereas in Europe this trend was reversed as the majority of identified data sources for surveillance were non-digital. This is illustrative of hospitals in the USA, which mostly have some form of electronic medical records.^19^

**Figure 3.**
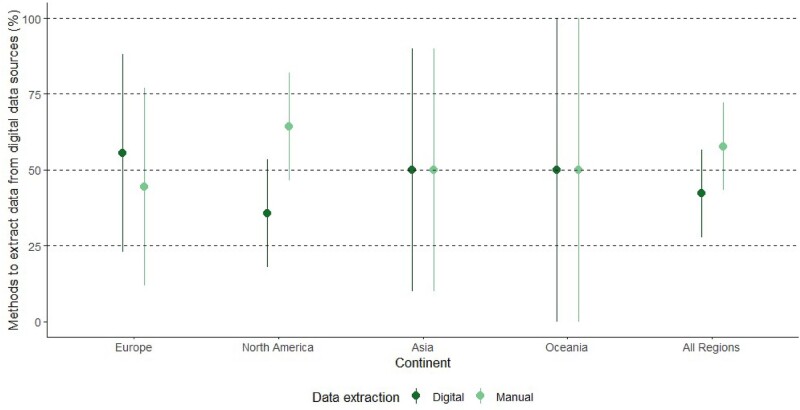
The types of data extraction methods employed by studies that harnessed data from digital sources for surveillance, by region. Europe, *n* = 9; North America, *n* = 28; Asia, *n* = 6; Oceania, *n* = 2; all regions, *n* = 45 (2 studies were unclear about method of data extraction). This figure appears in colour in the online version of *JAC* and in black and white in the printed version of *JAC*.

However, investigation of the method used to extract data from digital data sources revealed that more studies employ manual rather than digital approaches (58%, 95% CI 43.3–72.2). For example, two-thirds of the 28 studies that used digital data sources in North America extracted data manually (Figure 3).

### Professionals involved in surveillance

Eighty-seven of the 93 studies that identified the professionals who carried out surveillance involved clinically trained staff (94%, 95% CI 88.6–98.5). Identified professionals most frequently engaged with manual data extraction for surveillance (84%, 95% CI 76.4–91.4) and less frequently with digital (4%, 95% CI 0.2–8.4) or a combination of manual and digital data extraction (10%, 95% CI 3.7–15.7). However, for reports that did not identify the types of professionals involved, a smaller proportion of surveillance was based on manual data extraction (44%, 95% CI 26.6–60.9) and a greater proportion was based on digital data extraction (38%, 95% CI 20.7–54.3) (Table 3).

**Table 3.** Professionals engaged with different data extraction pipelines for surveillance

Data collector^a^, *n* = 145	Manual data extraction *n* (%, 95% CI)	Digital data extraction *n* (%, 95% CI)	Manual and digital *n* (%, 95% CI)	Not stated *n* (%, 95% CI)
Physician (± one other), *n* = 38	31 (82, 69.3–94.0)	1 (3, 0.0–7.7)	4 (11, 0.8–20.3)	2 (5, 0.0–12.4)
Clinical pharmacist (± one other), *n*** = **41	32 (78, 65.4–90.7)	1 (2, 0.0–7.2)	7 (17, 5.6–28.6)	1 (2, 0.0–7.2)
Nurse (± one other), *n*** = **9	9 (100)	0	0	0
Microbiologist (± one other), *n*** = **2	2 (100)	0	0	0
Laboratory (± one other), *n*** = **1	0	1 (100)	0	0
Pharmacy technician (± one other), *n*** = **1	1 (100)	0	0	0
Researcher (± one other), *n*** = **10	8 (80, 55.2–100)	1 (10, 0.0–28.6)	1 (10, 0.0–28.6)	0
Government body (± one other), *n*** = **1	1 (100)	0	0	0
AMS team, infection control team or a collaboration of at least three different clinical professions (e.g. nurse, physician, pharmacist), *n*** = **14	13 (93, 79.4–100)	1 (7, 0.0–20.6)	0	0
Unknown hospital staff, *n*** = **20	14 (70, 49.9–90.1)	3 (15, 0.0–30.7)	3 (15, 0.0–30.7)	0
Not stated, *n*** = **32	14 (44, 26.6–60.9)	12 (38, 20.7–54.3)	1 (3, 0.0–9.2)	5 (16, 3.1–28.2)

a Studies with several data collectors are counted as >1.

## Discussion

This review describes the data sources, data extraction methods and professionals involved in AMU surveillance approaches in the high-income secondary care setting. The results indicate that digital datasets are widely available for AMU surveillance in North America, but remain less commonly available in Europe. In regions where digitally available datasets are used for surveillance, stakeholders are still often relying on extracting these data manually from electronic systems. Healthcare professionals appear to be most frequently carrying out this work, but as the need for more targeted AMS is recognized, this labour-intensive model is likely to conflict with clinical responsibilities and could lead to missed opportunities if informatics skills are unavailable to harness digital datasets collected through increasingly prevalent EHRs.

### Data sources for surveillance

The concentration of studies that used electronic data sources for AMU surveillance in North America (28/47 studies) was likely determined by areas of healthcare policy not immediately related to AMR. The healthcare system in the USA is largely based on private healthcare providers generally afforded to citizens through employer-funded private health insurance and a minority of federal and state health insurance programmes.^20^ This system has been based on charging software for several decades. Combined with policies including financial incentives under the Health Information Technology for Economic and Clinical Health (HITECH) Act to promote the adoption of health information technology, this digitization has now extended to the wide roll-out of electronic prescribing and medical records in secondary care.^21–23^ In 2015, it was estimated that 84% of non-federal hospitals implemented at least a basic EHR including patient demographics, problem and medication lists, discharge summaries, lab and radiology reports and diagnostic test results.^19^ A later survey in 2017 estimated that 94% of hospitals harness data from these systems, most commonly for quality improvement (QI) purposes (82%).^24^ This trend of insurance structures for healthcare driving digitization of patient care is also observable in South Korea, where the national Health Insurance Review and Assessment (HIRA) service requests claims to be submitted in digital form, which has driven the adoption of computerized physician-order entry systems.^19^^,^^25^ By contrast, the majority of data sources identified in European surveillance were non-digital (27/38 studies for which the data source could be identified). Europe has had greater variability in uptake of digital systems in hospitals, with earlier uptake in Nordic countries but inconsistencies in functionality across the continent.^26^^,^^27^ Globally, digital patient care systems that passively collect data through clinical care *should* reduce the time and resources required to monitor patterns of prescribing compared with manual systems for surveillance.

### Data extraction for surveillance

In this review, digital records for patient care did not always enable stakeholders to electronically monitor AMU. Almost a fifth of studies included in this review manually extracted data from digital data sources for AMU surveillance (18%) (Table 2). Regions that implemented a lot of surveillance based on digital data sources were also regions that implemented high proportions of manual data extraction pipelines from digital data sources (Figure 3). Although high-income countries are broadly investing in electronic systems to improve patient care, based on the studies reported in the literature, they do not appear to be set up to facilitate QI and research.^28^ To support AMS most effectively, datasets on prescribing linked to others such as pathology and diagnostic codes to extract patient-level information on disease severity, clinical progression and outcomes are needed. However, barriers to the analysis of EHR data include: difficulties establishing pathways to extract and link data from a variety of patient care systems that are not interoperable; a lack of in-hospital expertise to analyse these datasets; and challenges navigating data protection guidelines to safely share these data with external stakeholders who have the expertise to support analysis for AMU audits and research.^29^ These kinds of ethical, legal, technical and professional skills challenges have all been cited previously in studies across England, Denmark, Switzerland and the USA.^30–34^

### Professionals involved in surveillance

The majority of surveillance captured in this review was undertaken by clinically trained professionals carrying out manual approaches to surveillance. This demands a high level of resource in terms of clinically trained time either taken away from patient care, or in addition to existing workload. For professionals with limited time, this conflict between manual surveillance of AMU and clinical responsibilities may become increasingly problematic as AMS stakeholders work towards achieving more detailed, continuous and coordinated surveillance. This model may also lead to missed opportunities requiring informatics skills to extract, link and harness datasets from patient care systems for surveillance. Unfortunately, a large proportion of the professionals involved in digital surveillance approaches could not be identified through surveillance reporting in this review. The need for dedicated, skilled professionals to facilitate the secondary use of datasets from electronic hospital systems for AMU surveillance is highlighted by the challenges with using EHRs already outlined. In the absence of a comprehensive and easy-to-use platform to access surveillance data, in-house hospital teams can improve access to datasets from EHRs, carry out analysis and advise external research teams regarding the navigation of ethics, data security and technological aspects of harnessing data from patient care systems at the hospital.

### Policy implications of this work

High-income countries are implementing national strategies to digitize patient care in hospitals and this infrastructure has the potential to replace labour-intensive models of manual surveillance of AMU. However, to do this, future policy must address the challenges with linking and harnessing datasets from these systems for AMS, as well as QI and research more broadly. This need for effective surveillance as part of AMS has been reiterated with urgency through high rates of antibiotic use among patients during the COVID-19 pandemic despite, so far, a low prevalence of secondary bacterial infection.^35^

### Limitations

The criterion for the exclusion of studies that did not report the total number of participants for whom AMU was estimated led to the exclusion of some national studies. This was implemented to ensure that the surveillance approach had been demonstrated in studies involving a large number of participants, as well as to include only approaches that provided denominator data as this is useful to adjust for when analysing AMU. A sensitivity analysis captured data extraction methods for these excluded national studies and found a higher proportion of these studies used digital surveillance approaches; however, these were not patient-level data sources. The data in this systematic review were extracted by a single reviewer (S.P.), but the data were extracted once and then checked in a second round of data extraction. Finally, no attempt was made to contact the authors of the studies included in this review so these results are based on study reporting only and a number of studies did not provide sufficient detail on the methods. The ability to understand the barriers to AMU surveillance from a systematic literature review is consequently limited. Qualitative studies would be valuable to explore the challenges identified in this review in more detail at local and national levels. For example, a large proportion of the professionals involved in digital surveillance approaches could not be identified in surveillance reporting captured in this review. This could represent either a separate type of professional not captured in this review, who is involved in surveillance and tends to drive digital surveillance approaches, or a type of identified professional who tends to be underreported in studies and is more frequently engaged in digital surveillance of AMU than described in these data.

### Conclusions

Electronic data sources for AMU surveillance are widely available in North America and remain less commonly available across Europe. However, surveillance in areas where electronic datasets are captured on AMU still most frequently employ manual methods to extract these data. This work is often carried out by healthcare professionals, which may conflict with clinical responsibilities. Future policy must address barriers to harnessing electronic datasets on AMU, which are already passively collected through clinical care or hospital administration, to progress with more effective AMS.

## Funding

S.P. receives funding for this work from the Economic and Social Research Council (ESRC; grant number ES/P000592/1). A.J. receives funding from the National Institute of Health Research (NIHR; grant number NIHR300293). S.H. was supported by funding from the National Institute for Health Research (NIHR) Health Protection Research Unit in Healthcare Associated Infections and Antimicrobial Resistance at the University of Oxford in partnership with Public Health England (NIHR200915). L.S. was funded by a National Institute for Health Research (NIHR) Clinician Scientist award (CS-2016-007) for this research project.

## Transparency declarations

None to declare. This publication presents independent research funded by the National Institute for Health Research (NIHR). The views expressed are those of the authors alone and not necessarily those of the NHS, the NIHR or the Department of Health and Social Care.

### Author contributions

S.P., A.J., L.S. and S.H. conceived the study topic and design. S.P. and A.J. carried out the study selection and data extraction. The data were analysed and the manuscript drafted by S.P. All authors contributed significantly to the revision of the manuscript and reviewed and approved the final version.

## Supplementary data

Tables S1 to S3 and Supplementary references are available as Supplementary data at *JAC* Online.

## Supplementary Material

dkab125_Supplementary_Data
