# Supplementary material for: Variation in approaches to antimicrobial use surveillance in high-income secondary care settings: a systematic review
Source: J Antimicrob Chemother. 2021 Apr 24;76(8):1969–77. doi: 10.1093/jac/dkab125 (PMC8283733; doi:10.1093/jac/dkab125)
Supplement: dkab125_Supplementary_Data [file dkab125_supplementary_data.docx]

# **Supplementary data**

Table S1. Websites searched to capture surveillance

|  | Organisation | Website |
| --- | --- | --- |
| 1 | Africa CDC: African Union | https://au.int/en/africacdc |
| 2 | Agency for Healthcare Research and Quality | www.ahrq.gov/ |
| 3 | Antibiotic Action | http://antibiotic-action.com/ |
| 4 | Alliance for the Prudent Use of Antibiotics (APUA) | www.apua.org/ |
| 5 | Australian Government Department of Health | www.health.gov.au/ |
| 6 | Australian Commission on Safety and Quality in Health Care \| Antimicrobial Use and Resistance in Australia (AURA) | https://www.safetyandquality.gov.au/antimicrobial-use-and-resistance-in-australia/ |
| 7 | British Society for Antimicrobial Chemotherapy | http://www.bsac.org.uk/ |
| 8 | British Infection Association | https://www.britishinfection.org/ |
| 9 | Center for Disease Dynamics, Economics & Policy (CDDEP) | https://www.cddep.org/  https://resistancemap.cddep.org/AntibioticUse.php |
| 10 | Canadian Foundation for Healthcare Improvement | https://www.cfhi-fcass.ca/ |
| 11 | Centers for Disease Control and Prevention (CDC) | https://www.cdc.gov/ |
| 12 | Care Quality Commission | https://www.cqc.org.uk/ |
| 13 | DANMAP | <https://www.danmap.org/> |
| 14 | European Centre for Disease Control and Prevention (ECDC) | https://ecdc.europa.eu/en/home |
| 15 | European Society of Clinical Microbiology and Infectious Diseases (ESCMID) | https://www.escmid.org/ |
| 16 | Global Point Prevalence Survey | <http://www.global-pps.com/> |
| 17 | Institute for Healthcare Improvement: Australian Council for Safety and Quality Health Care | http://www.ihi.org/resources/Pages/OtherWebsites/AustralianCouncilforSafetyandQualityinHealthCare.aspx |
| 18 | International Society of Chemotherapy Infection and Cancer (ISC) | www.ischemo.org/ |
| 19 | International Society of Infectious Diseases | www.isid.org/ |
| 20 | National Institute for Health and Care Excellence (NICE) | https://www.nice.org.uk/ |
| 21 | National Quality Measures Clearinghouse | https://www.qualitymeasures.ahrq.gov/ |
| 22 | NHS Improvement | https://improvement.nhs.uk/ |
| 23 | Public Health Agency of Canada | www.publichealth.gc.ca/ |
| 24 | Public Health England | <https://www.gov.uk/government/organisations/public-health-england>  https://fingertips.phe.org.uk/profile/amr-local-indicators |
| 25 | Public Health Surveillance: New Zealand Ministry of Health & Institute of Environmental Science Research Ltd (ESR) | https://surv.esr.cri.nz/index.php |
| 26 | RAND Corporation | www.rand.org/ |
| 27 | ReAct group | www.reactgroup.org/ |
| 28 | Swedish Strategic Programme against Antibiotic Resistance (STRAMA) | www.strama.se |
| 29 | Transatlantic Task Force on Antimicrobial Resistance (TATFAR) | *http://www.cdc.gov/drugresistance/tatfar/* |
| 30 | US Food and Drug Administration | www.fda.gov/ |
| 31 | World Health Organization | [www.who.int/](http://www.who.int/)  <http://www.afro.who.int/>  <https://www.paho.org/hq/>  <http://www.searo.who.int/en/>  <http://www.euro.who.int/en/home>  http://www.emro.who.int/index.html  http://www.wpro.who.int/en/ |
| 32 | Scottish Antimicrobial Prescribing Group | <http://www.isdscotland.org/Health-Topics/Prescribing-and-Medicines/SAPG/> |
| 33 | Health Protection Scotland | <https://www.hps.scot.nhs.uk/> |

Table S2. Surveillance characteristics

| **Author** | **Year** | **Study design** | **Country / Territory** | **Hospital type** |
| --- | --- | --- | --- | --- |
| Akhloufi H,Streefkerk R H,Melles D C,de Steenwinkel,J et al.^1^ | 2015 | cross-sectional | Netherlands | Tertiary hospital |
| al Harbi M^2^ | 1998 | cross-sectional | Saudi Arabia | unclear |
| Aldeyab M A,Kearney M P,McElnay J et al.^3^ | 2011 | cross-sectional | Northern Ireland | Teaching hospital |
| Aldeyab MA,Kearney MP,McElnay JC,et al.^4^ | 2012 | cross-sectional | Northern Ireland | multiple sites |
| Alfandari S,Robert J, Péan Y,et al.^5^ | 2015 | cross-sectional | France | multiple sites |
| Al-Ghamdi S,Gedebou M,Bilal N E^6^ | 2002 | cohort | Saudi Arabia | Primary / Secondary care hospital |
| Metsini A,Vazquez M,Sommerstein R et al.^7^ | 2018 | cross-sectional | Switzerland | multiple sites |
| Al-Somai N,Al-Muhur M,Quteimat O et al.^8^ | 2014 | quasi-experimental | Saudi Arabia | Tertiary hospital |
| Al-Taani G M,Scott M,Farren D,et al.^9^ | 2018 | cross-sectional | Northern Ireland | multiple sites |
| Al-Yamani A,Khamis F, Al-Zakwani I et al.^10^ | 2016 | cross-sectional | Oman | Tertiary hospital |
| Ansari F,Erntell M,Goossens H et al.^11^ | 2009 | cross-sectional | England,Scotland,Estonia,Sweden,Denmark,Netherlands,Northern Ireland,France,Austria,Belgium,Croatia,Czech Republic,Finland,Greece,Latvia,Lithuania,Malta,Norway,Poland,Slovenia | multiple sites |
| Armstrong E P,Kopp D L^12^ | 1985 | cross-sectional | USA | Primary / Secondary care hospital |
| Arnold FW,McDonald LC,Mangino PD et al.^13^ | 2004 | cross-sectional | USA | Teaching hospital |
| Berild D,Ringertz S H,Lelek M^14^ | 2002 | cross-sectional | Norway | Teaching hospital |
| Blakiston M,Zaman S^15^ | 2014 | cross-sectional | New Zealand | Primary / Secondary care hospital |
| Boots R J,Lipman J,Bellomo R et al.^16^ | 2005 | cross-sectional | New Zealand,Australia | multiple sites |
| Bosch C M,Hulscher M E,Natsch S et al.^17^ | 2016 | cross-sectional | Netherlands | multiple sites |
| Braykov N P,Morgan D J,Schweizer Marin L et al.^18^ | 2014 | cohort | USA | multiple sites |
| Brownridge D J,Zaidi S T.R^19^ | 2017 | cross-sectional | Australia | unclear |
| Buess M,Schilter D,Schneider T et al.^20^ | 2017 | cohort | Switzerland | multiple sites |
| Burston J,Adhikari S,Hayen A et al.^21^ | 2017 | quasi-experimental | Australia | Tertiary hospital |
| Cairns S,Gibbons C,Milne A et al.^22^ | 2018 | cross-sectional | Scotland | multiple sites |
| Caplinger C,Smith G,Remington R et al.^23^ | 2016 | quasi-experimental | USA | Teaching hospital |
| Carruthers M M,Grant K^24^ | 1978 | cross-sectional | USA | Veterans hospital |
| Chalmers J D,Singanayagam A,Akram A R et al.^25^ | 2011 | quasi-experimental | Scotland | Teaching hospital |
| Chaves N J,Ingram R J,Macisaac C M et al.^26^ | 2014 | quasi-experimental | Australia | Teaching hospital |
| Cheng C,Lee C,Wu M,Chang et al.^27^ | 2016 | cohort | Taiwan | Tertiary hospital |
| Choe P G,Koo H L,Lee E et al.^28^ | 2018 | quasi-experimental | Korea | Teaching hospital |
| Seaton RA,Nathwani D,Phillips G et al.^29^ | 1999 | quasi-experimental | Scotland | multiple sites |
| Coleman R W,Rodondi L C,Kaubisch S et al.^30^ | 1991 | quasi-experimental | USA | Veterans hospital |
| Cooke D M,Salter A J,Phillips I^31^ | 1983 | cross-sectional | England | Teaching hospital |
| Cotta M O,Robertson M S,Upjohn L M et al.^32^ | 2014 | cross-sectional | Australia | multiple sites |
| Cotta M O,Chen C,Tacey M et al.^33^ | 2016 | cross-sectional | Australia | multiple sites |
| Covington E W,Eure S,Carroll D et al.^34^ | 2018 | quasi-experimental | USA | Primary / Secondary care hospital |
| de With K,Bestehorn H,Steib-Bauert M et al.^35^ | 2009 | cross-sectional | Germany | Teaching hospital |
| Delory T,De Pontfarcy,A,Emirian A et al.^36^ | 2013 | quasi-experimental | France | Teaching hospital |
| Deuster S,Roten I,Muehlebach S^37^ | 2010 | quasi-experimental | Switzerland | Teaching hospital |
| Diasinos N,Baysari M,Kumar S et al.^38^ | 2015 | cross-sectional | Australia | Teaching hospital |
| DiazGranados Carlos A^39^ | 2012 | quasi-experimental | USA | Teaching hospital |
| DiDiodato G,McArthur L,Beyene J et al.^40^ | 2016 | quasi-experimental | Canada | Teaching hospital |
| DiDiodato G,McArthur L^41^ | 2016 | quasi-experimental | Canada | Teaching hospital |
| Dimina E, Kūla M,Caune U et al.^42^ | 2009 | cross-sectional | Latvia | multiple sites |
| Dunn K,O'Reilly A,Silke B et al.^43^ | 2011 | quasi-experimental | Rep of Ireland | Teaching hospital |
| Étienne P,Roger P M,Brofferio P et al.^44^ | 2011 | cross-sectional | France | multiple sites |
| Chen C,McNeese-Smith D,Cowan M et al.^45^ | 2009 | quasi-experimental | USA | Teaching hospital |
| Cosgrove S E,Seo S K,Bolon M K et al.^46^ | 2012 | quasi-experimental | USA | multiple sites |
| Evans R S,Larsen R A,Burke J P^47^ | 1986 | cross-sectional | USA | Teaching hospital |
| Evans R S,Pestotnik S L,Classen D C et al.^48^ | 1998 | quasi-experimental | USA | Teaching hospital |
| Ezebuenyi M C,Brakta F,Onor I O et al.^49^ | 2018 | cross-sectional | USA | Primary / Secondary care hospital |
| Först G,de With K,Weber N et al.^50^ | 2017 | cross-sectional | Germany | multiple sites |
| Fournier A,Eggimann P,Pagani J et al.^51^ | 2015 | quasi-experimental | Switzerland | Tertiary hospital |
| Fowler S,Webber A,Cooper B S et al.^52^ | 2007 | quasi-experimental | England | Teaching hospital |
| Fukuda T,Watanabe H,Ido S et al.^53^ | 2014 | quasi-experimental | Japan | Tertiary hospital |
| Fusier I,Parent de Curzon,O,Touratier S et al.^54^ | 2017 | cross-sectional | France | multiple sites |
| Gastmeier P,Sohr D,Forster D et al.^55^ | 2000 | cross-sectional | Germany | multiple sites |
| Gendel I,Azzam Z S,Braun E,Levy Y et al.^56^ | 2004 | cross-sectional | Israel | Teaching hospital |
| Gilbert K,Gleason P P,Singer D E et al.^57^ | 1998 | cohort | USA,Canada | multiple sites |
| Glowacki R C,Schwartz D N,Itokazu G S et al.^58^ | 2003 | cross-sectional | USA | Teaching hospital |
| Grasela T H Jr,Welage L S,Walawander C A et al.^59^ | 1990 | cohort | USA,Canada | multiple sites |
| Greenlaw C W^60^ | 1977 | cross-sectional | USA | unclear |
| Grill E,Weber A,Lohmann S et al.^61^ | 2011 | quasi-experimental | Germany | Teaching hospital |
| Güerri-Fernández R, Villar-García J, Herrera-Fernández S et al.^62^ | 2016 | quasi-experimental | Spain | Teaching hospital |
| Gyssens I C,Kullberg B J^63^ | 1995 | quasi-experimental | Netherlands | Teaching hospital |
| Haas M K,Dalton K,Knepper B C et al.^64^ | 2016 | quasi-experimental | USA | Teaching hospital |
| Hammerman A,Greenberg A,Yinnon A M^65^ | 1997 | quasi-experimental | Israel | Teaching hospital |
| Hansen S,Sohr D,Piening B et al.^66^ | 2013 | cross-sectional | Germany | multiple sites |
| Hartley S E,Kuhn L,Valley S et al.^67^ | 2016 | quasi-experimental | USA | multiple sites |
| Herfindal E T,Bernstein L R,Kishi D T^68^ | 1983 | quasi-experimental | USA | multiple sites |
| Hirschhorn L R,Currier J S,Platt R^69^ | 1993 | cohort | USA | Teaching hospital |
| Hogli J U,Garcia B H,Skjold F et al.^70^ | 2016 | quasi-experimental | Norway | Teaching hospital |
| Hohmann C,Eickhoff C,Radziwill R et al.^71^ | 2012 | cross-sectional | Germany | multiple sites |
| Ingram P R,Seet J M,Budgeon C A et al.^72^ | 2012 | cross-sectional | Australia | Tertiary hospital |
| Irfan N,Brooks A,Mithoowani S et al.^73^ | 2015 | quasi-experimental | Canada | multiple sites |
| Jones S R,Pannell J,Barks J et al.^74^ | 1977 | quasi-experimental | USA | Teaching hospital |
| Jozefiak E T,Lewicki J E,Kozinn W P^75^ | 1995 | cohort | USA | Teaching hospital |
| Kanerva M,Ollgren J,Lyytikainen O et al.^76^ | 2007 | cross-sectional | Finland | multiple sites |
| Karlsson M,Nilsson S O,Ransjo U^77^ | 1987 | cross-sectional | Sweden | unclear |
| Kern WV,Rose AD,Hay B et al.^78^ | 2001 | cross-sectional | Germany | multiple sites |
| Baek-Nam K^79^ | 2005 | quasi-experimental | Korea | Teaching hospital |
| Knox M C,Edye M^80^ | 2016 | quasi-experimental | Australia | Teaching hospital |
| Lacombe K,Cariou S,Tilleul P et al.^81^ | 2005 | quasi-experimental | France | Teaching hospital |
| Laing R B,Mackenzie A R,Shaw H et al.^82^ | 1998 | quasi-experimental | Scotland | unclear |
| Larsen R A,Curtis E T,Jacobson J A et al.^83^ | 1987 | cross-sectional | USA | Teaching hospital |
| Latorraca R,Martins R^84^ | 1979 | cross-sectional | USA | unclear |
| Lim C L.L,Lee W,Lee A L.C et al.^85^ | 2013 | cohort | Singapore | unclear |
| Lowe C F,Payne M,Puddicombe D et al.^86^ | 2017 | quasi-experimental | Canada | multiple sites |
| Magill S S,Edwards J R,Beldavs Z G et al.^87^ | 2014 | cross-sectional | USA | multiple sites |
| Malcolm W,Nathwani D,Davey P et al.^88^ | 2013 | quasi-experimental | Scotland | multiple sites |
| Mandy B,Koutny E,Cornette C et al.^89^ | 2004 | cross-sectional | France | Teaching hospital |
| Manuel O,Burnand B,Bady P et al.^90^ | 2010 | quasi-experimental | Canada | Teaching hospital |
| McConachy K A, Cuell, S, Kent PJ^91^ | 1999 | cross-sectional | Australia | unclear |
| McLellan L,Dornan T,Newton P et al.^92^ | 2016 | randomised controlled trial | England | Teaching hospital |
| Mehta J M,Haynes K,Wileyto E P et al.^93^ | 2014 | quasi-experimental | USA | Teaching hospital |
| Metcalfe J,Lam A,Lam S S H et al.^94^ | 2017 | quasi-experimental | Australia | Teaching hospital |
| Meyer E,Lapatschek M,Bechtold A et. al^95^ | 2009 | quasi-experimental | Germany | Teaching hospital |
| Mol P G M,Wieringa J E,Nannanpanday P V et al.^96^ | 2005 | quasi-experimental | Netherlands | Teaching hospital |
| Morioka H,Nagao M,Yoshihara S et al.^97^ | 2018 | cross-sectional | Japan | multiple sites |
| Moss F,McNicol M W,McSwiggan D A et al.^98^ | 1981 | cross-sectional | England | Primary / Secondary care hospital |
| Nagel J L,Huang A M,Kunapuli A et al.^99^ | 2014 | quasi-experimental | USA | Teaching hospital |
| Nault V,Pepin J,Beaudoin M et al.^100^ | 2017 | quasi-experimental | Canada | Teaching hospital |
| Elligsen M, Walker S A N, Simor A et al.^101^ | 2012 | quasi-experimental | Canada | Tertiary hospital |
| Ng CK,Wu TC,Chan WM et al.^102^ | 2008 | quasi-experimental | Hong Kong | Tertiary hospital |
| Nguyen C T,Gandhi T,Chenoweth C et al.^103^ | 2015 | quasi-experimental | USA | Teaching hospital |
| O'Neill E,Morris-Downes M,Rajan L et al.^104^ | 2010 | cross-sectional | Rep of Ireland | Tertiary hospital |
| Palmay L,Elligsen M,Walker S A et al.^105^ | 2014 | randomised controlled trial | Canada | Teaching hospital |
| Pastel D A,Chang S,Nessim S et al.^106^ | 1992 | quasi-experimental | USA | Teaching hospital |
| Mical P,Andreassen S,Tacconelli E et al.^107^ | 2006 | randomised controlled trial | Germany,Israel,Italy | multiple sites |
| Plumridge R J,McGechie D B^108^ | 1984 | cross-sectional | Australia | Teaching hospital |
| Popovski Z,Mercuri M,Main C et al.^109^ | 2015 | quasi-experimental | Canada | Teaching hospital |
| Porretta A,Giuliani L,Vegni F E et al.^110^ | 2003 | cross-sectional | Italy | multiple sites |
| Raineri E,Pan A,Mondello P et al.^111^ | 2008 | quasi-experimental | Italy | unclear |
| Remschmidt C,Behnke M,Kola A et al.^112^ | 2017 | ecological | Germany | Teaching hospital |
| Ritchie S,Scanlon N,Lewis M et al.^113^ | 2004 | quasi-experimental | New Zealand | multiple sites |
| Robert J, Péan Y,Varon E et al.^114^ | 2012 | cross-sectional | France | multiple sites |
| Roshdy D,Jaffa R,Pillinger K E et al.^115^ | 2018 | quasi-experimental | USA | Teaching hospital |
| Schön T,Sandelin L L,Bonnedahl J et al.^116^ | 2011 | quasi-experimental | Sweden | multiple sites |
| Semret M,Schiller I,Jardin B A et al.^117^ | 2017 | quasi-experimental | Canada | Teaching hospital |
| Branche A R,Walsh E E,Vargas R et al.^118^ | 2015 | randomised controlled trial | USA | Tertiary hospital |
| Skoog G,Struwe J,Cars O et al.^119^ | 2016 | cross-sectional | Sweden | multiple sites |
| So M,Mamdani M M,Morris A M et al.^120^ | 2018 | quasi-experimental | Canada | multiple sites |
| Stefkovicova M,Litvova S,Melus V et al.^121^ | 2016 | cross-sectional | Slovakia | multiple sites |
| Stevens G P,Jacobson J A,Burke J P^122^ | 1981 | cross-sectional | USA | Teaching hospital |
| Swearingen S M,White C,Weidert S et al.^123^ | 2016 | quasi-experimental | USA | Teaching hospital |
| Taggart Linda R,Leung Elizabeth,Muller M et al.^124^ | 2015 | quasi-experimental | Canada | Teaching hospital |
| Tavares M,Carvalho A C,Almeida J P et al.^125^ | 2018 | quasi-experimental | Portugal | Teaching hospital |
| Thuong M,Shortgen F,Zazempa V et al.^126^ | 2000 | quasi-experimental | France | Teaching hospital |
| Vaisman A,McCready J,Hicks S et al.^127^ | 2017 | quasi-experimental | Canada | Teaching hospital |
| van Kasteren,M E E,Kullberg B J,de Boer,A S et al.^128^ | 2003 | cross-sectional | Netherlands | multiple sites |
| van Spreuwel,P C J M,Blok H,Langelaar M F M et al.^129^ | 2015 | cross-sectional | Netherlands | Teaching hospital |
| Versporten A,Zarb P,Caniaux I et al.^130^ | 2018 | cross-sectional | England,Wales,Scotland,USA,Germany,Netherlands,Northern Ireland,France,Switzerland,Belgium,Finland,Latvia,Lithuania,New Zealand,Australia,Canada,Rep of Ireland | multiple sites |
| Von Seggern R L^131^ | 1987 | quasi-experimental | USA | Primary / Secondary care hospital |
| Wasserfallen J,Butschi A,Muff P et al.^132^ | 2004 | quasi-experimental | Switzerland | Teaching hospital |
| Willemsen I,Bogaers-Hofman D,Winters M et al.^133^ | 2009 | cross-sectional | Netherlands | Teaching hospital |
| Willemsen I,van der Kooij,T,van Benthem,B et al.^134^ | 2010 | cross-sectional | Netherlands | multiple sites |
| Witte K W,Nelson Jr A.A,Hutchinson R A^135^ | 1980 | quasi-experimental | USA | Teaching hospital |
| Wong S,Santullo P,Hirani S P et al.^136^ | 2017 | cross-sectional | England,Wales,Scotland,Netherlands,Belgium,Spain | multiple sites |
| Yamashita S K,Louie M,Simor A E et al.^137^ | 2000 | cross-sectional | Canada | Teaching hospital |
| Zahar J,Rioux C,Girou E et al.^138^ | 2006 | quasi-experimental | France | Teaching hospital |
| Zarkotou O,Avgoulea K,Papagiannakopoulou P et al.^139^ | 2016 | cross-sectional | Greece | Tertiary hospital |
| Ziółkowski G,Pawlowska I,Krawczyk L et al.^140^ | 2018 | ecological | Poland | Teaching hospital |
| Zoutman D,Chau L,Watterson J et al.^141^ | 1999 | cohort | Canada | multiple sites |
| Elhajji F D, Al-Taani G M, Anani L et al.^142^ | 2018 | cross-sectional | Northern Ireland | Teaching hospital |
| Tamma P D, Advic E, Li D X et al.^143^ | 2017 | cohort | USA | Teaching hospital |
| Health Protection Scotland ^144^ | 2017 | cross-sectional | Scotland | multiple sites |
| Filice G A, Drekonja D M, Thurn J Ret al.^145^ | 2015 | cohort | USA | Veterans hospital |

| Table S3. Surveillance approaches   \| **Author** \| **Data Source** \| **Data Extraction Method** \| **Professionals involved in data collection** \| \| --- \| --- \| --- \| --- \| \| Akhloufi H,Streefkerk R H,Melles D C,de Steenwinkel,J et al.^1^ \| Digital \| Digital \| Hospital staff \| \| al Harbi M^2^ \| Digital \| Digital \| Physician \| \| Aldeyab M A,Kearney M P,McElnay J et al.^3^ \| Paper \| Manual \| Pharmacist \| \| Aldeyab MA,Kearney MP,McElnay JC,et al.^4^ \| Paper \| Manual \| Pharmacist \| \| Alfandari S,Robert J,Pean Y,et al.^5^ \| Unclear \| Manual \| Unclear \| \| Al-Ghamdi S,Gedebou M,Bilal N E^6^ \| Unclear \| Manual \| Nurse \| \| Metsini A,Vazquez M,Sommerstein R et al.^7^ \| Unclear \| Manual \| Team \| \| Al-Somai N,Al-Muhur M,Quteimat O et al.^8^ \| Unclear \| Manual \| Pharmacist \| \| Al-Taani G M,Scott M,Farren D,et al.^9^ \| Unclear \| Manual \| Pharmacist \| \| Al-Yamani A,Khamis F,Al-Abri S et al.^10^ \| Digital \| Manual \| Hospital staff \| \| Ansari F,Erntell M,Goossens H et al.^11^ \| Unclear \| Manual \| Hospital staff \| \| Armstrong E P,Kopp D L^12^ \| Paper \| Manual \| Pharmacist, Researcher \| \| Arnold FW,McDonald LC,Mangino PD et al.^13^ \| Unclear \| Manual \| Unclear \| \| Berild D,Ringertz S H,Lelek M^14^ \| Unclear \| Manual \| Physician, Nurse \| \| Blakiston M,Zaman S^15^ \| Unclear \| Manual \| Government \| \| Boots R J,Lipman J,Bellomo R et al.^16^ \| Unclear \| Manual \| Hospital staff \| \| Bosch C M,Hulscher M E,Natsch S et al.^17^ \| Digital / Paper across different sites \| Manual \| Physician, Nurse \| \| Braykov N P,Morgan D J,Schweizer Marin L et al.^18^ \| Digital / Paper across different sites \| Manual \| Physician \| \| Brownridge D J,Zaidi S T.R^19^ \| Unclear \| Manual \| Pharmacist, Researcher \| \| Buess M,Schilter D,Schneider T et al.^20^ \| Unclear \| Manual \| Physician \| \| Burston J,Adhikari S,Hayen A et al.^21^ \| Combination \| Manual \| Physician, Researcher \| \| Cairns S,Gibbons C,Milne A et al.^22^ \| Combination \| Manual \| Team \| \| Caplinger C,Smith G,Remington R et al.^23^ \| Digital \| Manual \| Unclear \| \| Carruthers M M,Grant K^24^ \| Paper \| Manual \| Physician, Nurse \| \| Chalmers J D,Singanayagam A,Akram A R et al.^25^ \| Unclear \| Unclear \| Unclear \| \| Chaves N J,Ingram R J,Macisaac C M et al.^26^ \| Paper \| Manual \| Researcher \| \| Cheng C,Lee C,Wu M,Chang et al.^27^ \| Unclear \| Unclear \| Unclear \| \| Choe P G,Koo H L,Lee E et al.^28^ \| Unclear \| Manual \| Pharmacist \| \| Seaton RA,Nathwani D,Phillips G et al.^29^ \| Paper \| Manual \| Team \| \| Coleman R W,Rodondi L C,Kaubisch S et al.^30^ \| Digital \| Digital \| Hospital staff \| \| Cooke D M,Salter A J,Phillips I^31^ \| Combination \| Manual \| Pharmacist, Microbiologist \| \| Cotta M O,Robertson M S,Upjohn L M et al.^32^ \| Unclear \| Manual \| Physician, Pharmacist \| \| Cotta M O,Chen C,Tacey M et al.^33^ \| Unclear \| Manual \| Team \| \| Covington E W,Eure S,Carroll D et al.^34^ \| Digital \| Manual \| Pharmacist \| \| de With K,Bestehorn H,Steib-Bauert M et al.^35^ \| Unclear \| Manual \| Hospital staff \| \| Delory T,De Pontfarcy,A,Emirian A et al.^36^ \| Digital \| Unclear \| Physician, Pharmacist \| \| Deuster S,Roten I,Muehlebach S^37^ \| Paper \| Manual \| Pharmacist \| \| Diasinos N,Baysari M,Kumar S et al.^38^ \| Digital \| Digital \| Unclear \| \| DiazGranados Carlos A^39^ \| Digital \| Manual \| Physician \| \| DiDiodato G,McArthur L,Beyene J et al.^40^ \| Digital \| Manual \| Physician, Pharmacist \| \| DiDiodato G,McArthur L^41^ \| Digital \| Combination \| Physician, Pharmacist \| \| Dimina E,Kula M,Caune U et al.^42^ \| Paper \| Manual \| Physician \| \| Dunn K,O'Reilly A,Silke B et al.^43^ \| Paper \| Manual \| Pharmacist \| \| Etienne P,Roger P M,Brofferio P et al.^44^ \| Digital / Paper across different sites \| Manual \| Physician \| \| Chen C,McNeese-Smith D,Cowan M et al.^45^ \| Digital \| Digital \| Unclear \| \| Cosgrove S E,Seo S K,Bolon M K et al.^46^ \| Unclear \| Manual \| Hospital staff \| \| Evans R S,Larsen R A,Burke J P^47^ \| Digital \| Digital \| Team \| \| Evans R S,Pestotnik S L,Classen D C et al.^48^ \| Digital \| Digital \| Unclear \| \| Ezebuenyi M C,Brakta F,Onor I O et al.^49^ \| Digital \| Manual \| Unclear \| \| Forst G,de With K,Weber N et al.^50^ \| Unclear \| Manual \| Team \| \| Fournier A,Eggimann P,Pagani J et al.^51^ \| Digital \| Combination \| Hospital staff \| \| Fowler S,Webber A,Cooper B S et al.^52^ \| Digital \| Unclear \| Physician \| \| Fukuda T,Watanabe H,Ido S et al.^53^ \| Digital \| Digital \| Pharmacist, laboratory staff \| \| Fusier I,Parent de Curzon,O,Touratier S et al.^54^ \| Combination \| Manual \| Pharmacist \| \| Gastmeier P,Sohr D,Forster D et al.^55^ \| Paper \| Manual \| Physician \| \| Gendel I,Azzam Z S,Braun E,Levy Y et al.^56^ \| Unclear \| Manual \| Unclear \| \| Gilbert K,Gleason P P,Singer D E et al.^57^ \| Unclear \| Manual \| Physician, Researcher \| \| Glowacki R C,Schwartz D N,Itokazu G S et al.^58^ \| Combination \| Combination \| Pharmacist \| \| Grasela T H Jr,Welage L S,Walawander C A et al.^59^ \| Unclear \| Manual \| Pharmacist \| \| Greenlaw C W^60^ \| Unclear \| Manual \| Pharmacist \| \| Grill E,Weber A,Lohmann S et al.^61^ \| Unclear \| Manual \| Pharmacist \| \| Guerri-Fernandez R,Villar-Garcia J,Herrera-Fernandez S et al.^62^ \| Unclear \| Manual \| Physician \| \| Gyssens I C,Kullberg B J^63^ \| Combination \| Manual \| Nurse, Pharmacy technician \| \| Haas M K,Dalton K,Knepper B C et al.^64^ \| Combination \| Manual \| Unclear \| \| Hammerman A,Greenberg A,Yinnon A M^65^ \| Paper \| Manual \| Unclear \| \| Hansen S,Sohr D,Piening B et al.^66^ \| Unclear \| Manual \| Team \| \| Hartley S E,Kuhn L,Valley S et al.^67^ \| Unclear \| Manual \| Physician \| \| Herfindal E T,Bernstein L R,Kishi D T^68^ \| Digital \| Combination \| Unclear \| \| Hirschhorn L R,Currier J S,Platt R^69^ \| Digital \| Combination \| Physician \| \| Hogli J U,Garcia B H,Skjold F et al.^70^ \| Combination \| Manual \| Unclear \| \| Hohmann C,Eickhoff C,Radziwill R et al.^71^ \| Unclear \| Manual \| Pharmacist \| \| Ingram P R,Seet J M,Budgeon C A et al.^72^ \| Unclear \| Manual \| Physician, Pharmacist \| \| Irfan N,Brooks A,Mithoowani S et al.^73^ \| Combination \| Manual \| Unclear \| \| Jones S R,Pannell J,Barks J et al.^74^ \| Unclear \| Manual \| Physician, Pharmacist \| \| Jozefiak E T,Lewicki J E,Kozinn W P^75^ \| Unclear \| Combination \| Pharmacist \| \| Kanerva M,Ollgren J,Lyytikainen O et al.^76^ \| Unclear \| Manual \| Nurse \| \| Karlsson M,Nilsson S O,Ransjo U^77^ \| Paper \| Manual \| Hospital staff \| \| Kern WV,Rose AD,Hay B et al.^78^ \| Combination \| Manual \| Physician \| \| Baek-Nam K^79^ \| Unclear \| Manual \| Researcher \| \| Knox M C,Edye M^80^ \| Combination \| Manual \| Hospital staff \| \| Lacombe K,Cariou S,Tilleul P et al.^81^ \| Unclear \| Manual \| Pharmacist \| \| Laing R B,Mackenzie A R,Shaw H et al.^82^ \| Paper \| Manual \| Unclear \| \| Larsen R A,Curtis E T,Jacobson J A et al.^83^ \| Paper \| Manual \| Physician \| \| Latorraca R,Martins R^84^ \| Paper \| Manual \| Hospital staff \| \| Lim C L.L,Lee W,Lee A L.C et al.^85^ \| Digital \| Combination \| Hospital staff \| \| Lowe C F,Payne M,Puddicombe D et al.^86^ \| Digital \| Manual \| Unclear \| \| Magill S S,Edwards J R,Beldavs Z G et al.^87^ \| Combination \| Manual \| Researcher \| \| Malcolm W,Nathwani D,Davey P et al.^88^ \| Paper \| Manual \| Team \| \| Mandy B,Koutny E,Cornette C et al.^89^ \| Combination \| Combination \| Pharmacist \| \| Manuel O,Burnand B,Bady P et al.^90^ \| Digital \| Combination \| Physician, Researcher \| \| McConachy K A^91^ \| Paper \| Manual \| Pharmacist, Nurse \| \| McLellan L,Dornan T,Newton P et al.^92^ \| Paper \| Manual \| Physician, Pharmacist \| \| Mehta J M,Haynes K,Wileyto E P et al.^93^ \| Digital \| Digital \| Unclear \| \| Metcalfe J,Lam A,Lam S S H et al.^94^ \| Digital \| Combination \| Hospital staff \| \| Meyer E,Lapatschek M,Bechtold A et. al^95^ \| Digital \| Digital \| Unclear \| \| Mol P G M,Wieringa J E,Nannanpanday P V et al.^96^ \| Combination \| Manual \| Unclear \| \| Morioka H,Nagao M,Yoshihara S et al.^97^ \| Digital \| Manual \| Team \| \| Moss F,McNicol M W,McSwiggan D A et al.^98^ \| Paper \| Manual \| Hospital staff \| \| Nagel J L,Huang A M,Kunapuli A et al.^99^ \| Unclear \| Manual \| Team \| \| Nault V,Pepin J,Beaudoin M et al.^100^ \| Digital \| Digital \| Unclear \| \| Elligsen M, Walker S A N, Simor A et al.^101^ \| Digital \| Digital \| Unclear \| \| Ng CK,Wu TC,Chan WM et al.^102^ \| Digital \| Digital \| Unclear \| \| Nguyen C T,Gandhi T,Chenoweth C et al.^103^ \| Unclear \| Unclear \| Unclear \| \| O'Neill E,Morris-Downes M,Rajan L et al.^104^ \| Unclear \| Manual \| Hospital staff \| \| Palmay L,Elligsen M,Walker S A et al.^105^ \| Digital \| Combination \| Pharmacist \| \| Pastel D A,Chang S,Nessim S et al.^106^ \| Unclear \| Manual \| Pharmacist \| \| Mical P,Andreassen S,Tacconelli E et al.^107^ \| Unclear \| Manual \| Unclear \| \| Plumridge R J,McGechie D B^108^ \| Paper \| Manual \| Pharmacist \| \| Popovski Z,Mercuri M,Main C et al.^109^ \| Digital \| Combination \| Physician, Pharmacist \| \| Porretta A,Giuliani L,Vegni F E et al.^110^ \| Unclear \| Manual \| Physician \| \| Raineri E,Pan A,Mondello P et al.^111^ \| Digital \| Manual \| Physician \| \| Remschmidt C,Behnke M,Kola A et al.^112^ \| Digital \| Digital \| Unclear \| \| Ritchie S,Scanlon N,Lewis M et al.^113^ \| Paper \| Manual \| Hospital staff \| \| Robert J,Pean Y,Varon E et al.^114^ \| Paper \| Manual \| Physician, Pharmacist \| \| Roshdy D,Jaffa R,Pillinger K E et al.^115^ \| Digital \| Manual \| Physician \| \| Schön T,Sandelin L L,Bonnedahl J et al.^116^ \| Unclear \| Manual \| Microbiologist \| \| Semret M,Schiller I,Jardin B A et al.^117^ \| Unclear \| Digital \| Researcher \| \| Branche A R,Walsh E E,Vargas R et al.^118^ \| Digital \| Manual \| Researcher \| \| Skoog G,Struwe J,Cars O et al.^119^ \| Unclear \| Manual \| Physician \| \| So M,Mamdani M M,Morris A M et al.^120^ \| Digital \| Digital \| Unclear \| \| Stefkovicova M,Litvova S,Melus V et al.^121^ \| Unclear \| Manual \| Unclear \| \| Stevens G P,Jacobson J A,Burke J P^122^ \| Paper \| Manual \| Physician \| \| Swearingen S M,White C,Weidert S et al.^123^ \| Digital \| Combination \| Pharmacist \| \| Taggart Linda R,Leung Elizabeth,Muller M et al.^124^ \| Digital \| Digital \| Hospital staff \| \| Tavares M,Carvalho A C,Almeida J P et al.^125^ \| Digital \| Digital \| Unclear \| \| Thuong M,Shortgen F,Zazempa V et al.^126^ \| Unclear \| Manual \| Physician \| \| Vaisman A,McCready J,Hicks S et al.^127^ \| Unclear \| Unclear \| Unclear \| \| van Kasteren,M E E,Kullberg B J,de Boer,A S et al.^128^ \| Unclear \| Manual \| Team \| \| van Spreuwel,P C J M,Blok H,Langelaar M F M et al.^129^ \| Combination \| Manual \| Team \| \| Versporten A,Zarb P,Caniaux I et al.^130^ \| Unclear \| Manual \| Team \| \| Von Seggern R L^131^ \| Paper \| Manual \| Pharmacist \| \| Wasserfallen J,Butschi A,Muff P et al.^132^ \| Paper \| Manual \| Pharmacist \| \| Willemsen I,Bogaers-Hofman D,Winters M et al.^133^ \| Unclear \| Manual \| Hospital staff \| \| Willemsen I,van der Kooij,T,van Benthem,B et al.^134^ \| Unclear \| Manual \| Physician, Nurse \| \| Witte K W,Nelson Jr A.A,Hutchinson R A^135^ \| Unclear \| Manual \| Pharmacist \| \| Wong S,Santullo P,Hirani S P et al.^136^ \| Unclear \| Manual \| Hospital staff \| \| Yamashita S K,Louie M,Simor A E et al.^137^ \| Paper \| Manual \| Hospital staff \| \| Zahar J,Rioux C,Girou E et al.^138^ \| Unclear \| Manual \| Physician \| \| Zarkotou O,Avgoulea K,Papagiannakopoulou P et al.^139^ \| Digital \| Digital \| Unclear \| \| Ziółkowski G,Pawlowska I,Krawczyk L et al.^140^ \| Unclear \| Unclear \| Unclear \| \| Zoutman D,Chau L,Watterson J et al.^141^ \| Paper \| Manual \| Physician \| \| Elhajji F D, Al-Taani G M, Anani L et al.^142^ \| Paper \| Manual \| Pharmacist \| \| Tamma P D, Advic E, Li D X et al.^143^ \| Digital \| Manual \| Physician, Pharmacist \| \| Health Protection Scotland ^144^ \| Combination \| Manual \| Team \| \| Filice G A, Drekonja D M, Thurn J Ret al.^145^ \| Digital \| Manual \| Pharmacist, Nurse \| |  |  |  |
| --- | --- | --- | --- | --- | --- | --- | --- | --- | --- | --- | --- | --- | --- | --- | --- | --- | --- | --- | --- | --- | --- | --- | --- | --- | --- | --- | --- | --- | --- | --- | --- | --- | --- | --- | --- | --- | --- | --- | --- | --- | --- | --- | --- | --- | --- | --- | --- | --- | --- | --- | --- | --- | --- | --- | --- | --- | --- | --- | --- | --- | --- | --- | --- | --- | --- | --- | --- | --- | --- | --- | --- | --- | --- | --- | --- | --- | --- | --- | --- | --- | --- | --- | --- | --- | --- | --- | --- | --- | --- | --- | --- | --- | --- | --- | --- | --- | --- | --- | --- | --- | --- | --- | --- | --- | --- | --- | --- | --- | --- | --- | --- | --- | --- | --- | --- | --- | --- | --- | --- | --- | --- | --- | --- | --- | --- | --- | --- | --- | --- | --- | --- | --- | --- | --- | --- | --- | --- | --- | --- | --- | --- | --- | --- | --- | --- | --- | --- | --- | --- | --- | --- | --- | --- | --- | --- | --- | --- | --- | --- | --- | --- | --- | --- | --- | --- | --- | --- | --- | --- | --- | --- | --- | --- | --- | --- | --- | --- | --- | --- | --- | --- | --- | --- | --- | --- | --- | --- | --- | --- | --- | --- | --- | --- | --- | --- | --- | --- | --- | --- | --- | --- | --- | --- | --- | --- | --- | --- | --- | --- | --- | --- | --- | --- | --- | --- | --- | --- | --- | --- | --- | --- | --- | --- | --- | --- | --- | --- | --- | --- | --- | --- | --- | --- | --- | --- | --- | --- | --- | --- | --- | --- | --- | --- | --- | --- | --- | --- | --- | --- | --- | --- | --- | --- | --- | --- | --- | --- | --- | --- | --- | --- | --- | --- | --- | --- | --- | --- | --- | --- | --- | --- | --- | --- | --- | --- | --- | --- | --- | --- | --- | --- | --- | --- | --- | --- | --- | --- | --- | --- | --- | --- | --- | --- | --- | --- | --- | --- | --- | --- | --- | --- | --- | --- | --- | --- | --- | --- | --- | --- | --- | --- | --- | --- | --- | --- | --- | --- | --- | --- | --- | --- | --- | --- | --- | --- | --- | --- | --- | --- | --- | --- | --- | --- | --- | --- | --- | --- | --- | --- | --- | --- | --- | --- | --- | --- | --- | --- | --- | --- | --- | --- | --- | --- | --- | --- | --- | --- | --- | --- | --- | --- | --- | --- | --- | --- | --- | --- | --- | --- | --- | --- | --- | --- | --- | --- | --- | --- | --- | --- | --- | --- | --- | --- | --- | --- | --- | --- | --- | --- | --- | --- | --- | --- | --- | --- | --- | --- | --- | --- | --- | --- | --- | --- | --- | --- | --- | --- | --- | --- | --- | --- | --- | --- | --- | --- | --- | --- | --- | --- | --- | --- | --- | --- | --- | --- | --- | --- | --- | --- | --- | --- | --- | --- | --- | --- | --- | --- | --- | --- | --- | --- | --- | --- | --- | --- | --- | --- | --- | --- | --- | --- | --- | --- | --- | --- | --- | --- | --- | --- | --- | --- | --- | --- | --- | --- | --- | --- | --- | --- | --- | --- | --- | --- | --- | --- | --- | --- | --- | --- | --- | --- | --- | --- | --- | --- | --- | --- | --- | --- | --- | --- | --- | --- | --- | --- | --- | --- | --- | --- | --- | --- | --- | --- | --- | --- | --- | --- | --- | --- | --- | --- | --- | --- | --- | --- | --- | --- | --- | --- | --- | --- | --- | --- | --- | --- | --- | --- | --- | --- | --- | --- | --- | --- | --- | --- | --- | --- | --- | --- | --- | --- | --- | --- | --- | --- | --- | --- | --- | --- | --- | --- | --- | --- | --- | --- | --- | --- | --- | --- | --- | --- | --- | --- | --- | --- | --- | --- | --- | --- | --- | --- | --- | --- | --- | --- | --- | --- | --- | --- | --- | --- | --- | --- | --- | --- | --- | --- |

## References

**1.** Akhloufi H, Streefkerk RH, Melles DC, *et al.* Point prevalence of appropriate antimicrobial therapy in a Dutch university hospital. *European journal of clinical microbiology & infectious diseases : official publication of the European Society of Clinical Microbiology* (2015) doi:10.1007/s10096-015-2398-6.

**2.** al Harbi M. Antimicrobial prophylactic practice in surgical patients. *East African medical journal* (1998).

**3.** Aldeyab MA, Kearney M, McElnay J, *et al.* A point prevalence survey of antibiotic prescriptions: benchmarking and patterns of use. *British Journal of Clinical Pharmacology* (2011) **71**, 293.

**4.** Aldeyab MA, Kearney MP, McElnay JC, *et al.* A point prevalence survey of antibiotic use in four acute-care teaching hospitals utilizing the European Surveillance of Antimicrobial Consumption (ESAC) audit tool. *Epidemiology & Infection* (2012) **140**, 1714–1720.

**5.** Alfandari S, Robert J, Péan Y, *et al.* Antibiotic use and good practice in 314 French hospitals: The 2010 SPA2 prevalence study. *Médecine et Maladies Infectieuses* (2015) **45**, 475–480.

**6.** Al-Ghamdi S, Gedebou M & Bilal NE. Nosocomial infections and misuse of antibiotics in a provincial community hospital, Saudi Arabia. *Journal of Hospital Infection* (2002) **50**, 115–121.

**7.** Metsini A, Vazquez M, Sommerstein R, *et al.* Point prevalence of healthcare-associated infections and antibiotic use in three large Swiss acute-care hospitals. *Swiss Medical Weekly* (2018) **148**, w14617.

**8.** Al-Somai N, Al-Muhur M, Quteimat O, *et al*. The impact of clinical pharmacist and ID intervention in rationalization of antimicrobial use. *Saudi Pharmaceutical Journal* (2014) **22**, 516–521.

**9.** Al-Taani GM, Scott M, Farren D, *et al.* Longitudinal point prevalence survey of antibacterial use in Northern Ireland using the European Surveillance of Antimicrobial Consumption (ESAC) PPS and Global-PPS tool. *Epidemiology & Infection* (2018) **146**, 985–990.

**10.** Al-Yamani A, Khamis F, Al-Zakwani I, *et al.* Patterns of antimicrobial prescribing in a tertiary care hospital in Oman. *Oman Med. J.* (2016) **31**, 35–39.

**11.** Ansari F, Erntell M, Goossens H, *et al*. The European Surveillance of Antimicrobial Consumption (ESAC) Point-Prevalence Survey of Antibacterial Use in 20 European Hospitals in 2006. *Clin Infect Dis* (2009) **49**, 1496–1504.

**12.** Armstrong E & Kopp DI. A survey of prophylactic antibiotic use for total joint replacements in a community hospital. *Drug intelligence & clinical pharmacy* (1985) **10**: 753-7.

**13.** Arnold FW, McDonald LC, Mangino PD, *et al*. The appropriateness of hospital antimicrobial use between medical and surgical specialties. *Formulary* (2004) **39**, 304–308.

**14.** Berild D, Ringertz SH & Lelek M. Appropriate Antibiotic Use According to Diagnoses and Bacteriological Findings: Report of 12 Point-prevalence Studies on Antibiotic Use in a University Hospital. *Scandinavian Journal of Infectious Diseases* (2002) **34**, 56–60.

**15.** Blakiston M, & Zaman S. Nosocomial bacteriuria in elderly inpatients may be leading to considerable antibiotic overuse: an audit of current management practice in a secondary level care hospital in New Zealand. *Infection and Drug Resistance* (2014) **7**, 301.

**16.** Boots RJ, Lipman J, Bellomo R, *et al*. The Spectrum of Practice in the Diagnosis and Management of Pneumonia in Patients Requiring Mechanical Ventilation. Australian and New Zealand Practice in Intensive Care (ANZPIC II). *Anaesth Intensive Care* (2005) **33**, 87–100.

**17.** van den Bosch CMA, Hulscher MEJL, Natsch S, *et al.* Applicability of generic quality indicators for appropriate antibiotic use in daily hospital practice: a cross-sectional point-prevalence multicenter study. *Clin. Microbiol. Infect.* (2016) **22**, 888.

**18.** Braykov NP, Morgan D, Schweizer M,  *et al.* Assessment of empirical antibiotic therapy optimisation in six hospitals: an observational cohort study. *The Lancet Infectious Diseases* (2014) **14**, 1220–1227.

**19.** Brownridge DJ & Zaidi STR. Retrospective audit of antimicrobial prescribing practices for acute exacerbations of chronic obstructive pulmonary diseases in a large regional hospital. *Journal of Clinical Pharmacy and Therapeutics* (2017) **42**, 301–305.

**20.** Buess M, Schilter D, Schneider T, *et al.* Treatment of COPD Exacerbation in Switzerland: Results and Recommendations of the European COPD Audit. *RES* (2017) **94**, 355–365.

**21.** Burston J, Adhikari S, Hayen A, *et al.* A Role for Antimicrobial Stewardship in Clinical Sepsis Pathways: a Prospective Interventional Study. *Infection Control & Hospital Epidemiology* (2017) **38**, 1032–1038.

**22.** Cairns S, Gibbons C, Milne A, *et al.* Results from the third Scottish National Prevalence Survey: is a population health approach now needed to prevent healthcare-associated infections? *Journal of Hospital Infection* (2018) **99**, 312–317.

**23.** Caplinger C, Smith G, Remington R. *et al*. Evaluation of a Computerized Decision Support Intervention to Decrease Use of Anti-Pseudomonal Carbapenems in Penicillin Allergic Patients. *Antibiotics* (2016) **5**, 7.

**24.** Carruthers MM & Grant K. A practical method of antimicrobial surveillance. *Health laboratory science* (1978) **15**, 44–9.

**25.** Chalmers JD,  Singanayagam A, Akram AR, *et al.* Safety and efficacy of CURB65-guided antibiotic therapy in community-acquired pneumonia. *J Antimicrob Chemother* (2011) **66**, 416–423.

**26.** Chaves NJ, Ingram RJ, MacIsaac CM, *et al*. Sticking to minimum standards: implementing antibiotic stewardship in intensive care. *Internal Medicine Journal* (2014) **44**, 1180–1187.

**27.** Cheng C-Y, Lee C-Y, Wu M-W, *et al.* Prospective antimicrobial audit and feedback did not decrease case fatality: Experiences from a hospital in northern Taiwan. *The Journal of Infection in Developing Countries* (2016) **10**, 395–399.

**28.** Choe PG, Koo HL, Yoon D, *et al.* Effect of an intervention targeting inappropriate continued empirical parenteral vancomycin use: a quasi-experimental study in a region of high MRSA prevalence. *BMC Infectious Diseases* (2018) **18**, 178.

**29.** Seaton RA, Nathwani D, Phillips G, *et al*. Clinical record keeping in patients receiving antibiotics in hospital. *Health Bulletin* (1999) **57**, 128–133.

**30.** Coleman RW, Rodondi LC, Kaubisch S, *et al*. Cost-effectiveness of prospective and continuous parenteral antibiotic control: Experience at the Palo Alto Veterans affairs medical center from 1987 to 1989. *The American Journal of Medicine* (1991) **90**, 439–444.

**31.** Cooke DM, Salter AJ & Phillips I. The impact of antibiotic policy on prescribing in a London Teaching Hospital A one–day prevalence survey as an indicator of antibiotic use. *J Antimicrob Chemother* (1983) **11**, 447–453.

**32.** Cotta MO, Robertson MS, Upjohn LM, *et al.* Using periodic point-prevalence surveys to assess appropriateness of antimicrobial prescribing in Australian private hospitals. *Internal Medicine Journal* (2014) **44**, 240–246.

**33.** Cotta MO, Chen C, Tacey M, *et al.* What are the similarities and differences in antimicrobial prescribing between Australian public and private hospitals? *Internal Medicine Journal* (2016) **46**, 1182–1188.

**34.** Covington EW, Eure S, Carroll D. *et al*. Impact of Procalcitonin Monitoring on Duration of Antibiotics in Patients With Sepsis and/or Pneumonia in a Community Hospital Setting. *J. Pharm. Technol.* (2018) **34**, 109–116.

**35.** de With K, Bestehorn H, Steib-Bauert M. *et al*. Comparison of Defined versus Recommended versus Prescribed Daily Doses for Measuring Hospital Antibiotic Consumption. *Infection* (2009) **37**, 349–352.

**36.** Delory T, De Pontfarcy A, Emirian A, *et al.* Impact of a program combining pre-authorization requirement and post-prescription review of carbapenems: an interrupted time-series analysis. *Eur J Clin Microbiol Infect Dis* (2013) **32**, 1599–1604.

**37.** Deuster S, Roten I & Muehlebach S. Implementation of treatment guidelines to support judicious use of antibiotic therapy. *Journal of Clinical Pharmacy and Therapeutics* (2010) **35**, 71–78.

**38.** Diasinos N, Baysari M, Kumar S. *et al*. Does the availability of therapeutic drug monitoring, computerised dose recommendation and prescribing decision support services promote compliance with national gentamicin prescribing guidelines? *Internal Medicine Journal* (2015) **45**, 55–62.

**39.** DiazGranados CA. Prospective audit for antimicrobial stewardship in intensive care: Impact on resistance and clinical outcomes. *American Journal of Infection Control* (2012) **40**, 526–529.

**40.** DiDiodato G, McArthur L, Beyene J, *et al*. Evaluating the impact of an antimicrobial stewardship program on the length of stay of immune-competent adult patients admitted to a hospital ward with a diagnosis of community-acquired pneumonia: A quasi-experimental study. *American Journal of Infection Control* (2016) **44**, e73–e79.

**41.** DiDiodato G & McArthur L. Evaluating the Effectiveness of an Antimicrobial Stewardship Program on Reducing the Incidence Rate of Healthcare-Associated Clostridium difficile Infection: A Non-Randomized, Stepped Wedge, Single-Site, Observational Study. *PLoS ONE* (2016) **11**, e0157671.

**42.** Dimiņa E, Kūla M, Caune U, *et al.* Repeated prevalence studies on antibiotic use in Latvia, 2003-2007. *Eurosurveillance* (2009) **14**, 19307.

**43.** Dunn K, O’Reilly A, Silke B, *et al*. Implementing a pharmacist-led sequential antimicrobial therapy strategy: a controlled before-and-after study. *Int J Clin Pharm* (2011) **33**, 208–214.

**44.** Étienne P, Roger P, Brofferio P, *et al.* Antimicrobial stewardship program and quality of antibiotic prescriptions. *Médecine et Maladies Infectieuses* (2011) **41**, 608–612.

**45.** Chen C, McNeese-Smith D, Cowan M, *et al*. Evaluation of a nurse practitioner-led care management model in reducing inpatient drug utilization and cost. *Nurs Econ* (2009) **27**, 160–168.

**46.** Cosgrove SE, Seo SK, Bolon MK, *et al.* Evaluation of postprescription review and feedback as a method of promoting rational antimicrobial use: a multicenter intervention. *Infection Control & Hospital Epidemiology* (2012) **33**, 374–380.

**47.** Evans RS, Larsen RA, Burke, J. P., *et al.* Computer Surveillance of Hospital-Acquired Infections and Antibiotic Use. *JAMA* (1986) **256**, 1007–1011.

**48.** Evans RS, Pestotnik SL, Classen, D. C., *et al.* A Computer-Assisted Management Program for Antibiotics and Other Antiinfective Agents. *N Engl J Med* (1998) **338**, 232–8.

**49.** Ezebuenyi MC, Brakta, F., Onor, I. O., *et al.* Evaluation of Physician Prescribing Patterns For Antibiotics in the Treatment of Nonnecrotizing Skin and Soft Tissue Infections. *P T* (2018) **43**, 287–292.

**50.** Först G, de With K, Weber N, *et al.* Validation of adapted daily dose definitions for hospital antibacterial drug use evaluation: A multicentre study. *J. Antimicrob. Chemother.* (2017) **72**, 2931–2937.

**51.** Fournier A, Eggiman P, Pagani J-L, *et al.* Impact of the introduction of real-time therapeutic drug monitoring on empirical doses of carbapenems in critically ill burn patients. *Burns* (2015) **41**, 956–968.

**52.** Fowler S, Webber A, Cooper BS, *et al.* Successful use of feedback to improve antibiotic prescribing and reduce Clostridium difficile infection: a controlled interrupted time series. *J Antimicrob Chemother* (2007) **59**, 990–995.

**53.** Fukuda T, Watanabe H, Ido S, & Shiragami M. Contribution of antimicrobial stewardship programs to reduction of antimicrobial therapy costs in community hospital with 429 Beds --before-after comparative two-year trial in Japan. *Journal of Pharmaceutical Policy and Practice* (2014) **7**, 10.

**54.** Fusier I, Parent de Curzon O, Touratier S, *et al.* Amoxicillin–clavulanic acid prescriptions at the Greater Paris University Hospitals (AP–HP). *Médecine et Maladies Infectieuses* (2017) **47**, 42–49.

**55.** Gastmeier P, Sohr D, Forster D, *et al.* Identifying Outliers of Antibiotic Usage in Prevalence Studies on Nosocomial Infections. *Infection Control & Hospital Epidemiology* (2000) **21**, 324–328.

**56.** Gendel I, Azzam ZS, Braun, E., *et al*. Antibiotic utilization prevalence: prospective comparison between two medical departments in a tertiary care university hospital. *Pharmacoepidemiology and Drug Safety* (2004) **13**, 735–739.

**57.** Gilbert K, Gleason P, Singer D, *et al.* Variations in Antimicrobial Use and Cost in More Than 2,000 Patients with Community-acquired Pneumonia. *The American Journal of Medicine* (1998) **104**, 17–27.

**58.** Glowacki RC, Schwartz DN, Itokazi GS, *et al.* Antibiotic Combinations with Redundant Antimicrobial Spectra: Clinical Epidemiology and Pilot Intervention of Computer-Assisted Surveillance. *Clin Infect Dis* (2003) **37**, 59–64.

**59.** Grasela TH, Welage LS, Walawander CA, *et al.* A nationwide survey of antibiotic prescribing patterns and clinical outcomes in patients with bacterial pneumonia. *DICP* (1990) **24**, 1220–1225.

**60.** Greenlaw CW. Antimicrobial drug use monitoring by a hospital pharmacy. *Am J Hosp Pharm* (1977) **34**, 835–838.

**61.** Grill E, Weber A, Lohmann S, *et al.* Effects of pharmaceutical counselling on antimicrobial use in surgical wards: intervention study with historical control group,. *Pharmacoepidemiology and Drug Safety* (2011) **20**, 739–746.

**62.** Güerri-Fernández R, Villar-García J, Herrera-Fernández S, *et al.* An antimicrobial stewardship program reduces antimicrobial therapy duration and hospital stay in surgical wards. *Rev Esp Quimioter* (2016) **29**, 119–121.

**63.** Gyssens IC & Kullberg BJ. Improving the quality of antimicrobial drug use can result in cost containment. *Pharm World Sci* (1995) **17**, 163–167.

**64.** Haas MK, Dalton K, Knepper BC, *et al.* Effects of a Syndrome-Specific Antibiotic Stewardship Intervention for Inpatient Community-Acquired Pneumonia. (2016) *Open Forum Infect Dis* **3**, ofw186.

**65.** Hammerman A, Greenberg A & Yinnon AM. Drug use evaluation of ciprofloxacin: impact of educational efforts on appropriateness of use. *Journal of Clinical Pharmacy and Therapeutics* (1997) **22**, 415–420.

**66.** Hansen, S., Sohr, D., Piening, B., *et al.* Antibiotic usage in German hospitals: results of the second national prevalence study. *J Antimicrob Chemother* **68**, 2934–2939 (2013).

**67.** Hartley SE, Kuhm L, Valley, S., *et al.* Evaluating a Hospitalist-Based Intervention to Decrease Unnecessary Antimicrobial Use in Patients With Asymptomatic Bacteriuria. *Infection Control & Hospital Epidemiology* (2016) **37**, 1044–1051.

**68.** Herfindal ET, Bernstein LR & Kishi DT. Effect of clinical pharmacy services on prescribing on an orthopedic unit. *Am J Hosp Pharm* (1983) **40**, 1945–1951.

**69.** Hirschhorn LR, Currier JS & Platt R. Electronic Surveillance of Antibiotic Exposure and Coded Discharge Diagnoses as Indicators of Postoperative Infection and Other Quality Assurance Measures. *Infection Control & Hospital Epidemiology* (1993) **14**, 21–28.

**70.** Høgli JU, Garcia BH, Skjold F, *et al*. An audit and feedback intervention study increased adherence to antibiotic prescribing guidelines at a Norwegian hospital. *BMC Infectious Diseases* (2016) **16**, 96.

**71.** Hohmann C, Eickhoff C, Radziwill R. *et al*. Adherence to guidelines for antibiotic prophylaxis in surgery patients in German hospitals: a multicentre evaluation involving pharmacy interns. *Infection* (2012) **40**, 131–137.

**72.** Ingram PR, Seet JM, Budgeon, C. A. *et al*. Point-prevalence study of inappropriate antibiotic use at a tertiary Australian hospital. *Internal Medicine Journal* (2012) **42**, 719–721.

**73.** Irfan, N., Brooks, A., Mithoowani, S., *et al.* A Controlled Quasi-Experimental Study of an Educational Intervention to Reduce the Unnecessary Use of Antimicrobials For Asymptomatic Bacteriuria. *PLOS ONE* (2015) **10**, e0132071.

**74.** Jones SR, Pannell J, Barks J, *et al.* The effect of an educational program upon hospital antibiotic use. *Am. J. Med. Sci.* (1977) **273**, 79–85.

**75.** Jozefiak ET, Lewicki JE & Kozinn WP. Computer-assisted antimicrobial surveillance in a community teaching hospital. *Am J Health Syst Pharm* (1995) **52**, 1536–1540.

**76.** Kanerva M, Ollgren J, Lyytikäinen O. *et al*. Antimicrobial use in Finnish acute care hospitals: data from national prevalence survey, 2005. *J Antimicrob Chemother* (2007) **60**, 440–444.

**77.** Karlsson M, Nilsson S-O. & Ransjö U. Antibiotic Usage in Surgery in a Large Teaching Hospital. *Scandinavian Journal of Infectious Diseases* (1987) **19**, 123–130.

**78.** Kern WV, Rose AD, Hay B, *et al.* Antimicrobial Expenditures and Usage at Four University Hospitals. *Infection* (2001) **29**, 127–137.

**79.** Baek-Nam K. Compliance with an infectious disease specialist’s advisory consultations on targeted antibiotic usage. *Journal of Infection and Chemotherapy* (2005) **11**, 84–88.

**80.** Knox MC & Edye M. Educational Antimicrobial Stewardship Intervention Ineffective in Changing Surgical Prophylactic Antibiotic Prescribing. *Surgical Infections* (2016) **17**, 224–228.

**81.** Lacombe K, Cariou S, Tilleul P, *et al*. Optimizing fluoroquinolone utilization in a public hospital: a prospective study of educational intervention. *Eur J Clin Microbiol Infect Dis* (2005) **24**, 6–11.

**82.** Laing RB, Mackenzie AR, Shaw, H., *et al*. The effect of intravenous-to-oral switch guidelines on the use of parenteral antimicrobials in medical wards. *J Antimicrob Chemother* (1998) **42**, 107–111.

**83.** Larsen RA, Curtis ET, Jacobson JA, *et al*. Trends in infections and antibiotic usage in a community hospital. *American Journal of Infection Control* (1987) **15**, 7–15.

**84.** Latorraca R. & Martins R. Surveillance of Antibiotic Use in a Community Hospital. *JAMA* (1979) **242**, 2585–2587.

**85.** Lim CL-L, Lee W, Lee AL-C, *et al.* Evaluation of Ertapenem use with Impact Assessment on Extended-Spectrum Beta-Lactamases (ESBL) Production and Gram-Negative resistance in Singapore General Hospital (SGH). *BMC Infectious Diseases* (2013) **13**, 523.

**86.** Lowe CF, Payne M, Puddicombe D, *et al.* Antimicrobial stewardship for hospitalized patients with viral respiratory tract infections. *American Journal of Infection Control* (2017) **45**, 872–875.

**87.** Magill SS, Edwards JR, Beldavs ZG, *et al.* Prevalence of Antimicrobial Use in US Acute Care Hospitals, May-September 2011. *JAMA* (2014) **312**, 1438–1446.

**88.** Malcolm W, Nathwani D, Davey P, *et al.* From intermittent antibiotic point prevalence surveys to quality improvement: experience in Scottish hospitals. *Antimicrobial Resistance and Infection Control* (2013) **2**, 3.

**89.** Mandy B, Koutny E, Cornette C, Woronoff-Lemsi M-C. & Talon, D. Methodological validation of monitoring indicators of antibiotics use in hospitals. *Pharm World Sci* (2004) **26**, 90–95.

**90.** Manuel O, Burnand B, Bady P, *et al.* Impact of standardised review of intravenous antibiotic therapy 72 hours after prescription in two internal medicine wards. *Journal of Hospital Infection* (2010) **74**, 326–331.

**91.** McConachy KA, Cuell S, Kent PJ *et al*. Surgical Antibiotic Prophylaxis in a Private Hospital Compliance with Guidelines. *The Australian Journal of Hospital Pharmacy* (1999) **29**, 5–9.

**92.** McLellan L, Dornan T, Newton P, *et al.* Pharmacist-led feedback workshops increase appropriate prescribing of antimicrobials. *J Antimicrob Chemother* (2016) **71**, 1415–1425.

**93.** Mehta JM, Haynes K, Wileyto PE, *et al.* Comparison of Prior Authorization and Prospective Audit with Feedback for Antimicrobial Stewardship. *Infect Control Hosp Epidemiol* (2014) **35**, 1092–1099.

**94.** Metcalfe J, Lam A, Lam SSH., *et al*. Impact of the introduction of computerised physician order entry (CPOE) on the surveillance of restricted antimicrobials and compliance with policy. *Journal of Pharmacy Practice and Research* (2017) **47**, 200–206.

**95.** Meyer E, Lapatschek M, Andreas B, *et al.* Impact of restriction of third generation cephalosporins on the burden of third generation cephalosporin resistant K. pneumoniae and E. coli in an ICU. *Intensive Care Med* (2009) **35**, 862–870.

**96.** Mol PGM, Wieringa JE, NannanPanday P, *et al.* Improving compliance with hospital antibiotic guidelines: a time-series intervention analysis. *J Antimicrob Chemother* (2005) **55**, 550–557.

**97.** Morioka H, Nagao M, Yoshihara S, *et al.* The first multi-centre point-prevalence survey in four Japanese university hospitals. *Journal of Hospital Infection* (2018) **99**, 325–331.

**98.** Moss F, Mcswiggan DA, Mcnicol MW & Miller DL. Survey of Antibiotic Prescribing in a District General Hospital I. Pattern of Use. *The Lancet* (1981) **318**, 349–352.

**99.** Nagel JL, Huang AM, Kunapuli, A., *et al.* Impact of Antimicrobial Stewardship Intervention on Coagulase-Negative Staphylococcus Blood Cultures in Conjunction with Rapid Diagnostic Testing. *Journal of Clinical Microbiology* (2014) **52**, 2849–2854.

**100.** Nault V, Pepin J, Beaudoin M, *et al.* Sustained impact of a computer-assisted antimicrobial stewardship intervention on antimicrobial use and length of stay. *J Antimicrob Chemother* (2017) **72**, 933–940.

**101.** Elligsen M, Walker SAN, Pinto R, *et al.* Audit and feedback to reduce broad-spectrum antibiotic use among intensive care unit patients: a controlled interrupted time series analysis. *Infect Control Hosp Epidemiol* (2012) **33**, 354–361.

**102.** Ng CK, Wu TC, Chan WMJ, *et al.* Clinical and economic impact of an antibiotics stewardship programme in a regional hospital in Hong Kong. *BMJ Quality & Safety* (2008) **17**, 387–392.

**103.** Nguyen C, Gandhi R, Chenoweth CT, *et al.* Impact of an antimicrobial stewardship-led intervention for Staphylococcus aureus bacteraemia: a quasi-experimental study. *J Antimicrob Chemother* (2015) **70**, 3390–3396.

**104.** O’Neill E, Morris-Downes M, Rajan L, *et al.* Combined audit of hospital antibiotic use and a prevalence survey of healthcare-associated infection. *Clinical Microbiology and Infection* (2010) **16**, 513–515.

**105.** Palmay L, Elligsen M, Walker S, *et al.* Hospital-wide Rollout of Antimicrobial Stewardship: A Stepped-Wedge Randomized Trial. *Clin Infect Dis* (2014) **59**, 867–874.

**106.** Pastel DA, Chang S, Nessim S, *et al*. Department of pharmacy-initiated program for streamlining empirical antibiotic therapy. *Hosp Pharm* (1992) **27**, 596–603, 614.

**107.** Mical P, Andreassen S, Tacconelli E, *et al.* Improving empirical antibiotic treatment using TREAT, a computerized decision support system: cluster randomized trial. *J Antimicrob Chemother* (2006) **58**, 1238–1245.

**108.** Plumridge RJ & McGechie DB. Assuring rational antibiotic use: the impact of a joint microbiology-pharmacy surveillance program. *Aust Health Rev* (1984) **7**, 269–277.

**109.** Popovski Z, Mercuri M, Main C, *et al.* Multifaceted intervention to optimize antibiotic use for intra-abdominal infections. *J Antimicrob Chemother* (2015) **70**, 1226–1229.

**110.** Porretta A, Guiliani L, Vegni FE, *et al.* Prevalence and patterns of antibiotic prescribing in Italian hospitals. *Infection* (2003) **31 Suppl 2**, 16–21.

**111.** Raineri E, Pan A, Mondello P, *et al.* Role of the infectious diseases specialist consultant on the appropriateness of antimicrobial therapy prescription in an intensive care unit. *Am J Infect Control* (2008) **36**, 283–290.

**112.** Remschmidt C, Behnke M, Kola A, *et al.* The effect of antibiotic use on prevalence of nosocomial vancomycin-resistant enterococci- an ecologic study. *Antimicrobial Resistance & Infection Control* (2017) **6**, 95.

**113.** Ritchie S, Scanlon N, Lewis M. *et al*. Use of a preprinted sticker to improve the prescribing of prophylactic antibiotics for hip fracture surgery. *Qual Saf Health Care* (2004) **13**, 384–387.

**114.** Robert J, Péan Y, Varon E, *et al.* Point prevalence survey of antibiotic use in French hospitals in 2009. *J Antimicrob Chemother* (2012) **67**, 1020–1026.

**115.** Roshdy D, Jaffa R, Pillinger KE, *et al.* Effect of a multifaceted stewardship intervention on antibiotic prescribing and outcomes for acute bacterial skin and skin structure infections: *Therapeutic Advances in Infectious Disease* (2018) **5**, 103 - 109

**116.** Schön T, Sandelin LL, Bonnedahl J, *et al.* A comparative study of three methods to evaluate an intervention to improve empirical antibiotic therapy for acute bacterial infections in hospitalized patients. *Scandinavian Journal of Infectious Diseases* (2011) **43**, 251–257.

**117.** Semret M, Schiller I, Jardin BA, *et al.* Multiplex Respiratory Virus Testing for Antimicrobial Stewardship: A Prospective Assessment of Antimicrobial Use and Clinical Outcomes Among Hospitalized Adults. *Journal of Infectious Diseases* (2017) **216**, 936–944.

**118.** Branche AR, Walsh EE, Vargas R,  *et al.* Serum Procalcitonin Measurement and Viral Testing to Guide Antibiotic Use for Respiratory Infections in Hospitalized Adults: A Randomized Controlled Trial. *Journal of Infectious Diseases* (2015) **212**, 1692–1700.

**119.** Skoog G, Struwer J, Cars O, *et al.* Repeated nationwide point-prevalence surveys of antimicrobial use in Swedish hospitals: data for actions 2003–2010. *Eurosurveillance* (2016) **21**, 30264.

**120.** So M, Mamdani MM, Morris AM, *et al.* Effect of an antimicrobial stewardship programme on antimicrobial utilisation and costs in patients with leukaemia: a retrospective controlled study. *Clinical Microbiology and Infection* (2018) **24**, 882–888.

**121.** Stefkovicova M, Litvova S, Melus V, *et al*. Point prevalence study of antimicrobial usage in acute care hospitals in the Slovak Republic. *J. Hosp. Infect.* **93**, 403–409 (2016).

**122.** Stevens GP, Jacobson JA & Burke JP. Changing Patterns of Hospital Infections and Antibiotic Use: Prevalence Surveys in a Community Hospital. *Arch Intern Med* (1981) **141**, 587–592.

**123.** Swearingen S, White C, Weidert S, *et al.* A multidimensional antimicrobial stewardship intervention targeting aztreonam use in patients with a reported penicillin allergy. *International Journal of Clinical Pharmacy* (2016) **38**, 213–217.

**124.** Taggart LR, Leung E, Muller MP, *et al*. Differential outcome of an antimicrobial stewardship audit and feedback program in two intensive care units: a controlled interrupted time series study. *BMC Infectious Diseases* (2015) **15**, 480.

**125.** Tavares M, Carvalho AC, Almeida JP, *et al.* Implementation and impact of an audit and feedback antimicrobial stewardship intervention in the orthopaedics department of a tertiary-care hospital: a controlled interrupted time series study. *International Journal of Antimicrobial Agents* (2018) **51**, 925–931.

**126.** Thuong M, Shortgen F, Zazempa V, *et al.* Appropriate use of restricted antimicrobial agents in hospitals: the importance of empirical therapy and assisted re-evaluation. *J Antimicrob Chemother* (2000) **46**, 501–508.

**127.** Vaisman A, McCready J, Hicks S, *et al*. Optimizing preoperative prophylaxis in patients with reported β-lactam allergy: a novel extension of antimicrobial stewardship. *J Antimicrob Chemother* (2017) **72**, 2657–2660.

**128.** van Kasteren MEE, Kullberg BJ, de Boer AS, *et al*. Adherence to local hospital guidelines for surgical antimicrobial prophylaxis: a multicentre audit in Dutch hospitals. *J Antimicrob Chemother* (2003) **51**, 1389–1396.

**129.** van Spreuwel PCJM, Blok H, Langelaar MFM, *et al.* Identifying targets for quality improvement in hospital antibiotic prescribing. *Neth J Med* (2015) **73**, 161–168.

**130.** Versporten A, Zarb P, Caniaux I, *et al.* Antimicrobial consumption and resistance in adult hospital inpatients in 53 countries: results of an internet-based global point prevalence survey. *The Lancet Global Health* (2018) **6**, e619–e629.

**131.** von Seggern RL. Culture and antibiotic monitoring service in a community hospital. *Am J Hosp Pharm* (1987) **44**, 1358–1362.

**132.** Wasserfallen J-B, Bütschi A-J, Muff P, *et al.* Format of medical order sheet improves security of antibiotics prescription: The experience of an intensive care unit*. *Critical Care Medicine* (2004) **32**, 655–659.

**133.** Willemsen I, Bogaers-Hofman D, Winters M. *et al*. Correlation between antibiotic use and resistance in a hospital: Temporary and ward-specific observations. *Infection* (2009) **37**, 432.

**134.** Willemsen I, Kooij T, van der Benthem B, *et al*. Appropriateness of antimicrobial therapy: a multicentre prevalence survey in the Netherlands, 2008–2009. *Eurosurveillance* (2010) **15**, 19715.

**135.** Witte KW, Nelson AA & Hutchinson RA. Effect of pharmacist consultation on rational antimicrobial therapy. *Am J Hosp Pharm* (1980) **37**, 829–832.

**136.** Wong S, Santullo P, Hirani SP, *et al.* Use of antibiotics and the prevalence of antibiotic-associated diarrhoea in patients with spinal cord injuries: an international, multi-centre study. *Journal of Hospital Infection* (2017) **97**, 146–152.

**137.** Yamashita SK, Louie M, Simor AE, *et al*. Microbiological surveillance and parenteral antibiotic use in a critical care unit. *Can J Infect Dis* (2000) **11**, 107–111.

**138.** Zahar J-R, Rioux C, Girou E, *et al.* Inappropriate prescribing of aminoglycosides: risk factors and impact of an antibiotic control team. *J Antimicrob Chemother* (2006) **58**, 651–656.

**139.** Zarkotou O, Avgoulea K, Papagiannakopoulou P,  *et al.* Five-year trends of antimicrobial drugs consumption and incidence of bloodstream infections caused by multidrug-resistant pathogens in a Greek ICU. *Acta Microbiologica Hellenica* (2016) **61**, 12.

**140.** Ziółkowski G, Pawłowska I, Krawczyk L, *et al*. Antibiotic consumption versus the prevalence of multidrug-resistant Acinetobacter baumannii and Clostridium difficile infections at an ICU from 2014–2015. *Journal of Infection and Public Health* (2018) **11**, 626–630.

**141.** Zoutman D, Chau L, Watterson J, *et al*. A Canadian Survey of Prophylactic Antibiotic Use Among Hip-Fracture Patients. *Infection Control & Hospital Epidemiology* (1999) **20**, 752–755.

**142.** Elhajji FD, Al-Taani GM, Anani L, *et al.* Comparative point prevalence survey of antimicrobial consumption between a hospital in Northern Ireland and a hospital in Jordan. *BMC Health Services Research* (2018) **18**, 849.

**143.** Tamma PD, Avdic E, Li DX, Dzintars, K. *et al*. Association of Adverse Events With Antibiotic Use in Hospitalized Patients. *JAMA Intern Med* (2017) **177**, 1308–1315.

**144.** Health Protection Scotland 2017. *National Point Prevalence Survey of Healthcare Associated Infection and Antimicrobial Prescribing 2016*. https://www.hps.scot.nhs.uk/web-resources-container/national-point-prevalence-survey-of-healthcare-associated-infection-and-antimicrobial-prescribing-2016/?id=3236 (2017).

**145.** Filice GA, Drekonja DM, Thurn JR, *et al.* Diagnostic Errors that Lead to Inappropriate Antimicrobial Use. *Infection Control & Hospital Epidemiology* (2015) **36**, 949–956.
